# Involvement of mitogen- and stress-activated protein kinase 1 in BMP-6–induced chondrocyte differentiation

**DOI:** 10.1016/j.jbc.2024.107806

**Published:** 2024-09-21

**Authors:** Naoko Nakano, Etsu Tashiro, Takayuki Shimada, Masayasu Ebisawa, Sayaka Kojima, Kaho Ayabe, Yohei Yamamoto, Shingo Maeda, Fumiko Itoh, Susumu Itoh

**Affiliations:** 1 Laboratory of Biochemistry, Showa Pharmaceutical University, Machida, Tokyo, Japan; 2 Department of Bone and Joint Medicine, Graduate School of Medical and Dental Sciences, Kagoshima University, Kagoshima, Kagoshima, Japan; 3 Laboratory of Stem Cell Regulation, Tokyo University of Pharmacy and Life Sciences, Hachioji, Tokyo, Japan

**Keywords:** ATDC5 cell, BMP, chondrocyte, MSK1, p38 kinase

## Abstract

Bone morphogenetic proteins (BMPs) are involved in several cellular responsive actions, such as development, cell differentiation, and apoptosis, *via* their specific transmembrane receptors. In particular, BMPs promote the differentiation and maturation of bone and cartilage from mesenchymal stem cells. Based on comprehensive analyses performed with a large number of antibodies, mitogen- and stress-activated protein kinase (MSK)1 was found to be immediately phosphorylated in the mouse chondrocyte precursor cell line, ATDC5, upon BMP-6 stimulation. The overexpression and knockdown of MSK1 in ATDC5 cells also enhanced and suppressed BMP-6–induced chondrocyte differentiation, respectively. Similar to ATDC5 cells, an *ex vivo* organ culture system using mouse embryonic metatarsal bones also demonstrated that BMP-6–mediated MSK1 activation might play a role in chondrocyte differentiation. Using several inhibitors, the p38 kinase pathway was confirmed to be implicated in BMP-6–induced phosphorylation of MSK1. Furthermore, MSK1 mutants lacking kinase activities and those lacking serine/threonine residues targeted by p38 kinase severely impaired their ability to potentiate BMP-6–induced chondrogenic differentiation of ATDC5 cells. Interestingly, a loss-of-function study for Smad4 perturbed BMP-6–induced phosphorylation of p38 kinase to inhibit BMP-6–mediated chondrocyte differentiation *via* MSK1 activation. Overall, both Smad-dependent and independent pathways require BMP-6–induced chondrocyte differentiation *via* MSK1 activation in ATDC5 cells.

Bone morphogenetic proteins (BMPs) are secreted polypeptides that play crucial roles in the formation and maintenance of many organs, including the cartilage, bone, muscle, and blood vessels (1). After BMPs bind to their specific type II receptors, including the BMP type II receptor, activin type II receptor, or activin type IIB receptor, the type II receptors can then interact with BMP type I receptors ((activin receptor–like kinase)2 [ALK2], ALK3, or ALK6) to phosphorylate their glycine-serine repeat domains in their juxtamembrane regions. Consequently, the intracellular serine/threonine kinase in BMP type I receptors becomes active, promoting the phosphorylation of the two extreme C-terminal serine residues of BMP receptor–regulated-Smad proteins, such as Smad1 and Smad5. The two phosphorylated BMP receptor–regulated-Smads make then form a complex with one Smad4 to enter the nucleus, where the ternary Smad complex regulates the transcription of BMP target genes together with other transcriptional factors, coactivators, and corepressors (1). Although Smad-dependent signaling is the main signal transduction pathway upon BMP receptor activation, cytoplasmic molecules other than Smads are also involved in BMP signaling, termed Smad-independent signaling pathway. In some cases, full activation of the BMP pathway is required for both Smad-dependent and independent signaling (2, 3, 4). The mitogen-activating protein (MAP) kinase pathways including the extracellular signal–regulated kinase (ERK), c-Jun-NH_2_-terminal kinase (JNK) and p38 kinase, PI3 kinase, and IκB kinase are well-known representatives of the Smad-independent pathway. These Smad-independent pathways are not common in all cell types, unlike the Smad-dependent pathway; therefore, the occurrence of the BMP-mediated Smad-independent pathway is context-dependent (3, 4).

Cartilage, which does not include nerves, blood vessels, and lymphoid vessels, consists of a single cell type, termed chondrocytes, and different extracellular matrix (ECM) proteins, such as highly sulfated proteoglycans. Mesenchymal stem cells (MSCs) are aggregated to become chondroprogenitor cells, they transform into chondrocytes. Chondrocytes develop into hypertrophic chondrocytes in several steps, which ultimately results in endochondral ossification. The fate of hypertrophic chondrocytes is not limited to apoptosis but also includes their transformation into osteoblasts or marrow MSCs during endochondral ossification. During chondrocyte maturation into hypertrophic chondrocytes, type X collagen, encoded by the *Col10a1* gene, is induced (5, 6, 7). Although several cytokines are known to contribute to the growth and differentiation of chondrocytes (7), BMPs can influence chondrocyte differentiation and bone differentiation because the deletion of both *Smad1* and *Smad5* genes in cartilage results in severe chondrodysplasia (8, 9).

Mitogen- and stress-activated protein kinase (MSK)1, a serine/threonine kinase, is ubiquitously expressed in many organs (10, 11). MSK1 shares approximately 40% similarity with ribosomal protein S6 kinases and comprises two kinase domains (12). MSK1 is downstream of the ERK and p38 kinase pathways. MSK1 possesses N-terminal and C-terminal kinase domains, which are members of the AGC family (13) and calmodulin-dependent protein kinase, respectively (14). After the C-terminal kinase of MSK1 is activated by ERK or p38 kinase, this protein is autophosphorylated. As a result, the N-terminal kinase in MSK1 becomes active (15, 16, 17). MSK1 is known to phosphorylate a number of substrates, such as histone H3, cAMP-response element-binding protein (CREB), activating transcription factor 1, NF-κB, ataxin 1, high-mobility group protein-14, RAR-related orphan receptor α, KDM3A, Trim7, and Trim28 (11, 16, 18, 19, 20, 21, 22). In addition, MSK1 has been reported to function as an effector of p38 kinase in the transforming growth factor (TGF-β) signaling pathway (23). TGF-β–induced cell death is antagonized by MSK1 because MSK1 influences proapoptotic BH3-only Bcl2 protein (24).

In the current study, we sought to understand how chondrocyte differentiation is regulated by BMP signaling. More than 50 types of antibodies were used for Western blot analyses to determine the protein(s) influenced by BMP signaling. We identified MSK1, which is immediately phosphorylated upon BMP-6 signaling. Gain- and loss-of-function studies revealed that MSK1 plays an important role in chondrocyte differentiation.

## Results

### Phosphorylation of MSK1 *via* the p38 kinase pathway upon BMP signaling in ATDC5 cells

**Figure 1:**
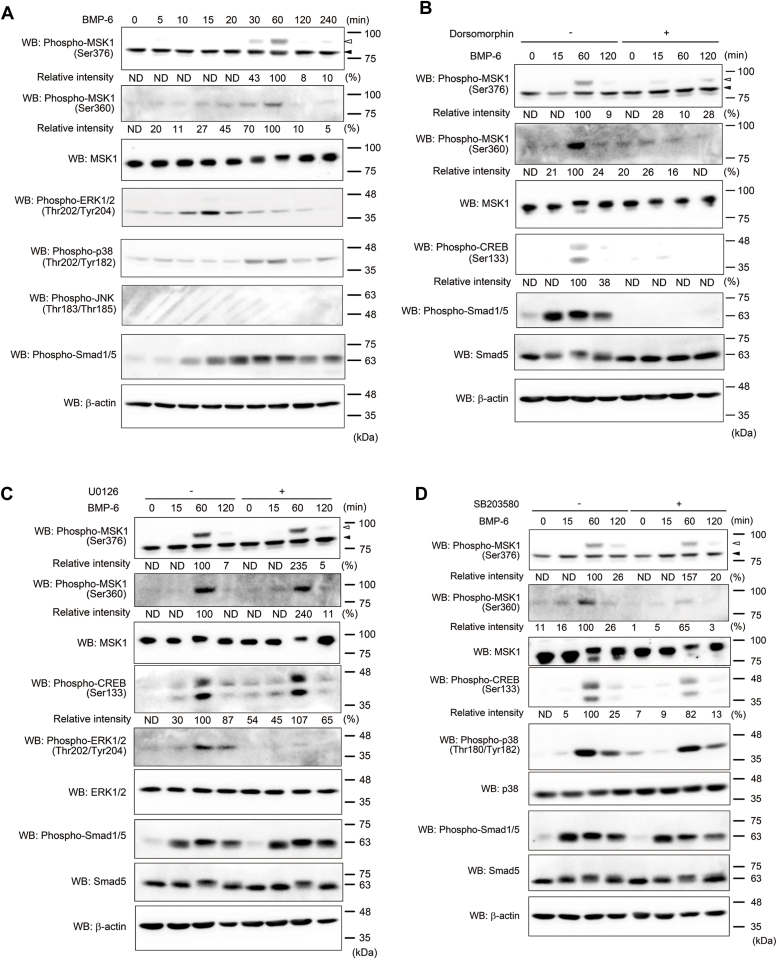

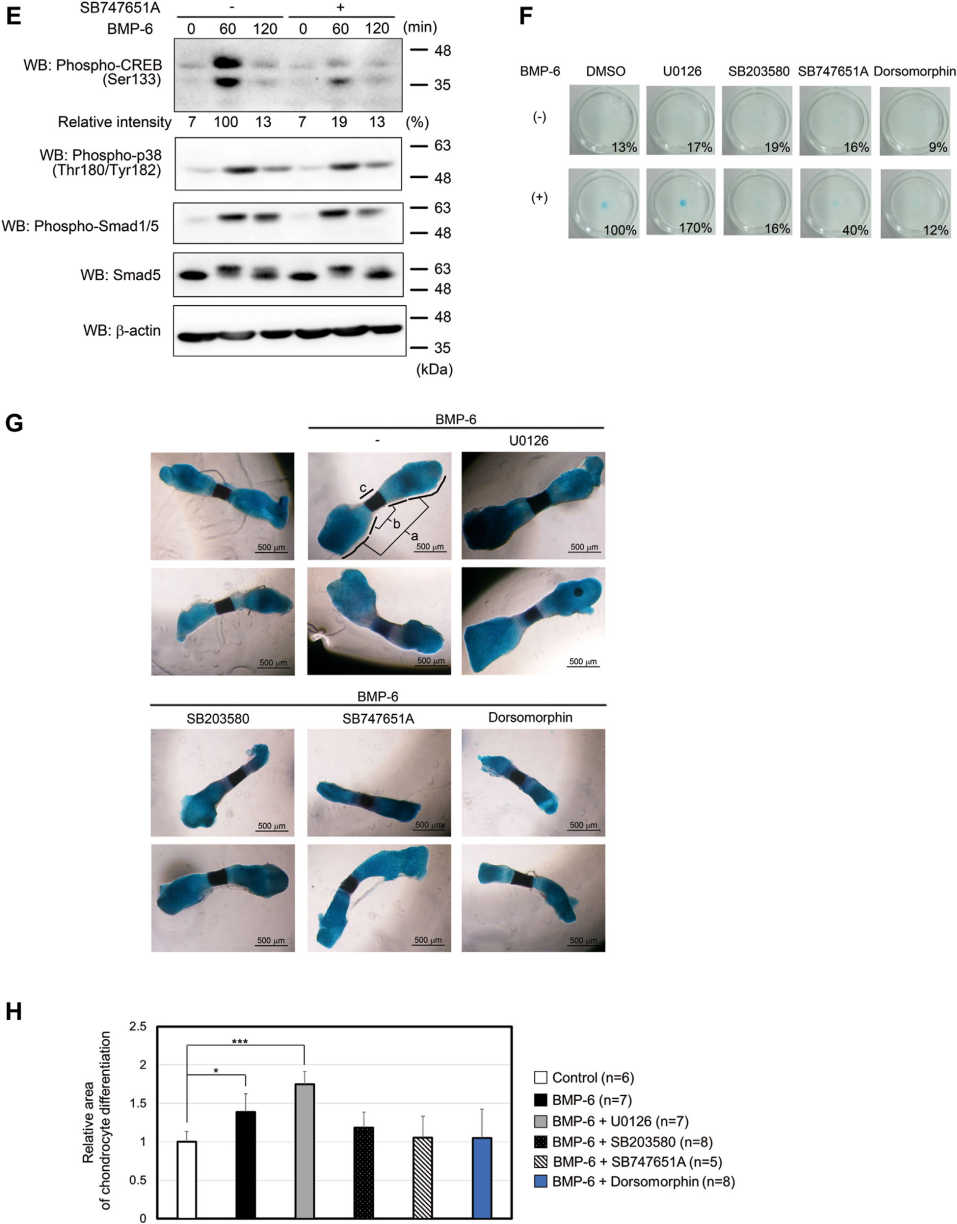
**Phosphorylation of MSK1 *via* the p38 kinase pathway upon BMP-6 stimulation in ATDC5 cells.***A*, phosphorylation of MSK1 (Ser376) and MSK1 (Ser360) by BMP-6. ATDC5 cells were stimulated with 25 ng/ml BMP-6 for the indicated times. The total cell lysates were then used for Western blot analyses. The total expression levels of phospho-MSK1 (Ser376), phospho-MSK1 (Ser360), MSK1, phospho-ERK1/2 (Thr202/Tyr204), phospho-p38 (Thr180/Tyr182), phospho-JNK (Thr183/Tyr185), phospho-Smad1/5, and β-actin are indicated in the *upper, second, third, fourth, fifth, sixth, seventh*, and *bottom panels*, respectively. The intensity of the bands for phospho-MSK1 (Ser376) and phospho-MSK1 (Ser360) was normalized to the intensity of the band corresponding to MSK1. Relative intensity was calculated with respect to the 1-h treatment of cells with BMP-6. *B*, Inhibition of BMP-6-induced MSK1 phosphorylation by dorsomorphin, a BMP type I receptor kinase inhibitor. ATDC5 cells were pretreated with 10 μM dorsomorphin for 1 h. Thereafter, cells were stimulated with 25 ng/ml BMP-6 for the indicated times. The total cell lysates were used for Western blot analyses. The total expression levels of phospho-MSK1 (Ser376), phospho-MSK1 (Ser360), MSK1, phospho-CREB (Ser133), phospho-Smad1/5, Smad5, and β-actin are indicated in the *upper, second, third, fourth, fifth, sixth*, and *bottom panels*, respectively. The intensity of the bands for phospho-MSK1 (Ser376) and phospho-MSK1 (Ser360) was normalized to the intensity of the band corresponding to MSK1. The intensity of the band for phospho-CREB (Ser133) was normalized to the intensity of the band corresponding to β-actin. Relative intensity was calculated with respect to the 1-h treatment of cells with BMP-6 in the absence of dorsomorphin. *C*, Involvement of the ERK pathway in the phosphorylation of MSK1 by BMP-6. ATDC5 cells were pretreated with 20 μM U0126 1 h before stimulation with 25 ng/ml BMP-6 for the indicated times. The total expression levels of phospho-MSK1 (Ser376), phospho-MSK1 (Ser360), MSK1, phospho-CREB (Ser133), phospho-ERK1/2 (Thr202/Tyr204), ERK1/2, phospho-Smad1/5, Smad5, and β-actin are indicated in the *upper, second, third, fourth, fifth, sixth, seventh, eighth*, and *bottom panels*, respectively. The intensity of the bands for phospho-MSK1 (Ser376) and phospho-MSK1 (Ser360) was normalized to the intensity of the band corresponding to MSK1. The intensity of the band for phospho-CREB (Ser133) was normalized to the intensity of the band corresponding to β-actin. Relative intensity was calculated with respect to the 1-h treatment of cells with BMP-6 in the absence of U0126. *D*, decrease in BMP-6-induced phosphorylation of MSK1 in the presence of the p38 kinase inhibitor. ATDC5 cells pretreated with 10 μM SB203580, a p38 kinase inhibitor, for 1 h were stimulated with 25 ng/ml BMP-6 for the indicated times. The total expression levels of phospho-MSK1 (Ser376), phospho-MSK1 (Ser360), MSK1, phospho-CREB (Ser133), phospho-p38 (Thr180/Tyr182), p38, phospho-Smad1/5, Smad5, and β-actin are indicated in the *upper, second, third, fourth, fifth, sixth, seventh, eighth*, and *bottom panels*, respectively. The intensity of the bands for phospho-MSK1 (Ser376) and phospho-MSK1 (Ser360) was normalized to the intensity of the band corresponding to MSK1. The intensity of the band for phospho-CREB (Ser133) was normalized to the intensity of the band corresponding to β-actin. Relative intensity was calculated with respect to the 1-h treatment of cells with BMP-6 in the absence of SB203580. *A-D*, *white and black arrowheads* indicate phospho-MSK1 (Ser376)-specific and nonspecific bands, respectively. *E*, decrease in BMP-6-mediated phospho-CREB (Ser133) expression by the MSK1 kinase inhibitor, SB747651A. ATDC5 cells were cultured with 10 μM SB747651A for 1 h. Prior to preparation of the total lysates, 25 ng/ml BMP-6 was added to the culture media for the indicated times. The total expression levels of phospho-CREB (Ser133), phospho-p38 (Thr180/Tyr182), phospho-Smad1/5, Smad5, and β-actin are indicated in the *upper, second, third, fourth*, and *bottom panels*, respectively. The intensity of the band for phospho-CREB (Ser133) was normalized to the intensity of the band corresponding to β-actin. Relative intensity was calculated with respect to the 1-h treatment of cells with BMP-6 in the absence of SB747651A. *F*, chondrogenic differentiation of ATDC5 cells by BMP-6 in the presence of kinase inhibitors. ATDC5 cells cultured at a high density were stimulated with 25 ng/ml BMP-6 in the presence (+) or absence (−) of kinase inhibitors (20 μM U0126, 10 μM SB203580, 10 μM SB747651A, or 10 μM dorsomorphin). Two weeks later, the cells were stained with Alcian blue. Representative images are shown. The intensity of the area stained with Alcian blue was measured using ImageQuant TL. The relative intensity of the stained area was calculated with respect to the 1-h treatment of cells with BMP-6 alone. *G*, representative images of *ex vivo* chondrogenic differentiation of mouse metatarsal bones induced by BMP-6 in the presence of kinase inhibitors. Each mouse metatarsal bone was cultured with or without 25 ng/ml BMP-6 for 7 days, either in the absence or presence of kinase inhibitors (20 μM U0126, 10 μM SB203580, 10 μM SB747651A, or 10 μM dorsomorphin). Then, each bone was stained with Alcian blue and Alizarin Red S. Two representative images are shown in each treatment. *a*, proliferative chondrocytes; *b*, clear zone represented as hypertrophic chondrocytes; *c*, chondrocyte matrix calcified by mature hypertrophic chondrocytes stained with alizarin red S. *H*, relative area of chondrocyte differentiation in mouse metatarsal bones induced by BMP-6 in the presence of kinase inhibitors. The total areas of chondrocyte differentiation (a + b + c) were measured. The total areas of chondrocyte differentiation (a + b + c) were measured using ImageJ. *Asterisks* indicate significant differences. BMP, bone morphogenetic protein; CREB, cAMP-response element-binding protein; MSK, mitogen- and stress-activated protein kinase; ND; not determined.

**Figure 2:**
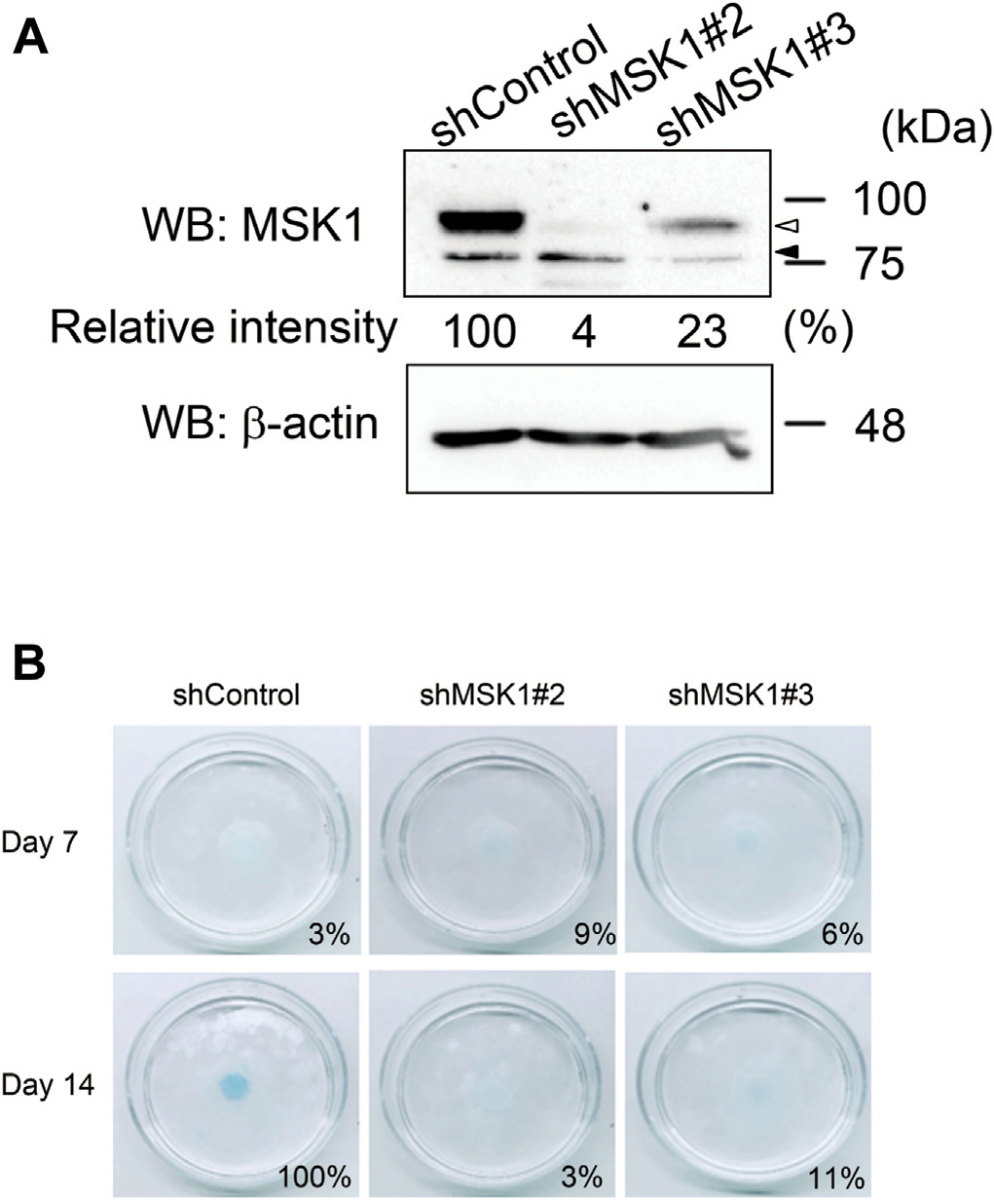
**Influence of MSK1 deficiency on BMP-6–mediated chondrogenic differentiation.***A*, expression of MSK1 in ATDC5 cells. Total lysates from each transformant were prepared for Western blot analyses. The total expression levels of MSK1 and β-actin are indicated in the *upper and lower panels*, respectively. *White and black arrowheads* indicate MSK1-specific and nonspecific bands, respectively. The intensity of the band for MSK1 was normalized to the intensity of the band corresponding to β-actin. Relative intensity was calculated with respect to cells carrying shControl. *B*, chondrogenic differentiation of ATDC5 cells with decreased expression of MSK1 in the presence of BMP-6. The cells were seeded at a high density and then stimulated with 25 ng/ml BMP-6. Seven (*upper panels*) or 14 days later (*lower panels*), the cells were stained with Alcian blue. Representative images are shown. The intensity of the area stained with Alcian blue was measured using ImageQuant TL. The relative intensity of the stained area was calculated with respect to the cells carrying shControl with BMP-6 for 14 days. BMP, bone morphogenetic protein; MSK, mitogen- and stress-activated protein kinase; qPCR, quantitative PCR.

BMP transduces intracellular signaling *via* Smad-dependent and independent pathways (3, 4). In particular, the Smad-independent pathways, termed the noncanonical BMP pathways, which include MAP kinases (ERK, JNK, and p38 kinase), PI3K, and IκB kinase, are activated in a context- and/or cell type–dependent manner (25). To identify the novel molecule(s) activated *via* the Smad-independent pathway in the chondrogenic cell line, ATDC5, we performed Western blot analyses using cell lysates of BMP-6–stimulated ATDC5 cells with more than 50 antibodies. Among these, anti-phospho-MSK1 (S376) and anti-phospho-MSK1 (Ser360) antibodies recognized specific bands in cell lysates from ATDC5 cells stimulated with 25 ng/ml BMP-6 for 30 and 60 min (Fig. 1*A*). As MSK1 is phosphorylated *via* the MAP kinase pathway including ERK, JNK and p38 kinase (12), we also examined the phosphorylation of these kinases upon BMP-6 stimulation. Both ERK and p38 kinase were phosphorylated by BMP-6 immediately before phosphorylation of MSK1, whereas BMP-6–mediated phosphorylation of JNK was not observed in ATDC5 cells (Fig. 1*A*). Notably, JNK could be phosphorylated in ATDC5 cells under osmotic stress (Fig. S1). Given that MSK1 and MSK2 belong to the same kinase family, we investigated whether BMP-6 also induces the phosphorylation of MSK2 in ATDC5 cells. As anticipated, MSK2 was rapidly phosphorylated following BMP-6 stimulation, similar to MSK1. However, the level of phosphorylated MSK2 was lower than that of phosphorylated MSK1 (Fig. S2). Furthermore, our loss-of-function study for MSK1 clearly demonstrated the suppression of BMP-6–mediated chondrocyte differentiation (Fig. 2*B*). Therefore, our current study primarily focused on the role of MSK1 in BMP-6–mediated chondrocyte differentiation. To demonstrate that the activation of BMP type I receptor kinases is required for the phosphorylation of MSK1, 10 μM dorsomorphin, an inhibitor of BMP type I receptor kinases, was added to ATDC5 cells 1 h before stimulation with BMP-6. As expected, the phosphorylation of MSK1 was markedly reduced, similar to the phosphorylation of Smad1/5 *via* the canonical BMP/Smad pathway. In addition, phosphorylation of CREB, a target protein phosphorylated by MSK1 (12), was perturbed in ATDC5 cells stimulated with BMP-6 in the presence of dorsomorphin (Fig. 1*B*). As BMP-6 promoted the phosphorylation of ERK and p38 kinase in ATDC5 cells, we examined the effects of U0126 (a MEK kinase inhibitor) and SB203580 (a p38 kinase inhibitor) on BMP-6–induced MSK1 phosphorylation. As shown in Fig. 1, *C* and *D*, SB203580 inhibited BMP-6-mediated MSK1 (Ser360) phosphorylation, unlike U0126, although the phosphorylation of MSK1 (Ser376) upon BMP-6 stimulation was marginally perturbed by SB203580 when cells were stimulated with BMP-6 for 2 h. Consistently, CREB phosphorylation decreased in ATDC5 cells stimulated with BMP-6 in the presence of SB203580 but not in the presence of U0126. SB747651A, an MSK1 kinase inhibitor, also interfered with CREB phosphorylation (Fig. 1*E*).

ATDC5 cells exhibited chondrogenic differentiation in the presence of BMPs when cultured under high-density condition (26, 27, 28). By examining the effects of the inhibitors on BMP-6–mediated chondrocyte differentiation, three other inhibitors, besides U0126, were found to suppress BMP-6–mediated chondrocyte differentiation (Fig. 1*F*). These results indicate that MSK1 phosphorylation *via* the p38 pathway activated by BMP-6 might play a key role in the chondrogenic differentiation of ATDC5 cells.

### Involvement of BMP-6–mediated MSK1 activation in chondrocyte differentiation in mouse bone organ culture

To further investigate the role of MSK1 activated by BMP-6, we utilized an *ex vivo* organ culture system using mouse embryonic metatarsal bones (Fig. 1*G*). This system closely mimics the complex chondrogenic process in a three-dimensional structure, preserving native cell–cell and cell–ECM interactions, as well as cellular signaling (29). MSCs start to gather together to form a dense cluster, a process known as mesenchymal condensation, followed by the beginning of chondrocyte differentiation. Subsequently, the committed chondrocytes begin to proliferate, becoming what are termed proliferative chondrocytes (Fig. 1*G* (a)). Proliferative chondrocytes are stained with Alcian blue due to the production of the ECM components such as chondroitin sulfate and proteoglycans that include sulfates. After a period of proliferation, the chondrocytes undergo hypertrophy, increasing in size and becoming hypertrophic chondrocytes, which are represented as a clear zone (Fig. 1*G* (b)). Finally, hypertrophic chondrocytes facilitating the calcification of the surrounding ECM, which is stained with Alizarin red S (Fig. 1*G* (c)). Thus, we measured the area of proliferative chondrocytes, uncalcified hypertrophic chondrocytes, and mature hypertrophic chondrocytes (a + b + c). BMP-6 significantly increased the areas of chondrocyte differentiation. However, this increase tended to be disrupted by dorsomorphin, SB203580, or SB747651A, while U0126 appeared to promote it rather than having no effect (Fig. S1*H*). It is unclear why U0126 promotes *ex vivo* chondrogenic differentiation differently from other inhibitors. This issue should be addressed in the future. These results further support the involvement of BMP-6–mediated MSK1 activation in chondrocyte differentiation.

### Requirement of kinase activities in MSK1 for the differentiation of ATDC5 cells

**Figure 3:**
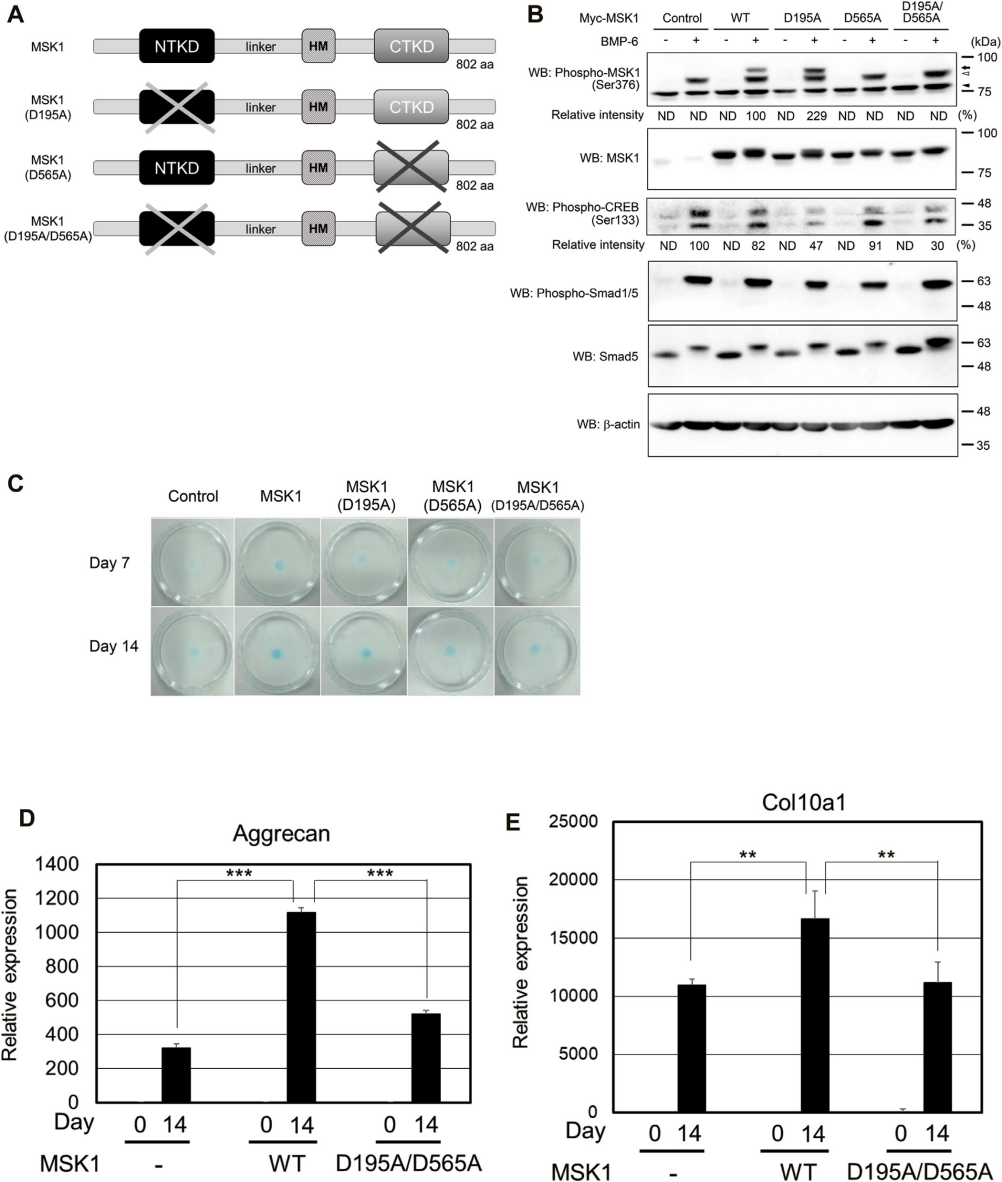
**Requirement of N-terminal and C-terminal kinase activities of MSK1 for the induction of chondrocyte differentiation.***A*, depiction of MSK1 and its mutants used in Figure 3. *B*, phosphorylation of MSK1 or its mutants in ATDC5 cells stimulated with BMP-6. To induce the expression of ectopic MSK1 or its mutants in ATDC5 cells, cells were cultured in the presence of tetracycline for 16 h. Thereafter, the ATDC5 transformants were stimulated with 25 ng/ml BMP-6 for 1 h. The total expression levels of phospho-MSK1 (Ser376), MSK1, phospho-CREB (Ser133), phospho-Smad1/5, Smad5, and β-actin are indicated in the *upper, second, third, fourth, fifth,* and *bottom panels*, respectively. A *black arrow and a white arrowhead* indicate ectopic and endogenous phospho-MSK1 (Ser376)-specific band, respectively. A *black arrowhead* indicates nonspecific bands. The intensities of the bands for phospho-MSK1 (Ser376) and phospho-CREB (Ser133) were normalized to the intensities of the bands corresponding to MSK1 and β-actin, respectively. The relative intensity was calculated with respect to the 1-h treatment of cells carrying WT MSK1 with BMP-6 alone. *C*, chondrogenic differentiation of ATDC5 cells carrying WT MSK1 or its mutants in the presence of BMP-6. After expression of WT MSK1 or its mutants in ATDC5 cells using tetracycline as described in Figure 3*B*, the cells were reseeded at a high density and stimulated with 25 ng/ml BMP-6. Seven (*upper photos*) or 14 days later (*lower photos*), the cells were stained with Alcian blue. Representative images are shown. The intensity of the area stained with Alcian blue was measured using ImageQuant TL. The relative intensity of the stained area was calculated with respect to the cells carrying WT MSK1 with BMP-6 alone for 14 days. *D* and *E*, According to Figure 3*C*, ATDC5 cells expressing WT MSK1 or MSK1 (D195A/D565A) were cultured with 25 ng/ml BMP-6 for 14 days. Thereafter, the total mRNAs from each transformant were prepared for qPCR to detect aggrecan (*D*) and collagen10a1 mRNAs (*E*). All values represent means ± SDs (n = 3). *Asterisks* indicate significant differences. BMP, bone morphogenetic protein; CTKD, C-terminal kinase domain; HM, hydrophobic motif; MSK, mitogen- and stress-activated protein kinase; ND, not determined; NTKD, N-terminal kinase domain; qPCR, quantitative PCR.

As MSK1 is a dual serine/threonine kinase (15, 30), we investigated whether both kinase activities are required for BMP-6–mediated chondrocyte differentiation. Briefly, we constructed three kinase-dead MSK1 mutants: MSK1 (D195A) lacking N-terminal kinase activity, MSK1 (D565A) lacking C-terminal kinase activity, and MSK1 (D195A/D565A) lacking both the N- and C-terminal kinases (Fig. 3*A*) (12). Thereafter, we adapted a tet-on expression system to induce the protein expression of MSK1 or its mutants because stable expression of MSK1 in ATDC5 cells may spontaneously promote the differentiation of ATDC5 cells. Sixteen hours after the addition of tetracycline to the cell culture media, the cells were stimulated with 25 ng/ml BMP-6 for 1 h. Thereafter, cell lysates were prepared for Western blot analysis. Cells expressing either the wild-type (WT) MSK1 or the MSK1 (D195A) mutant, both of which exhibit C-terminal kinase activity, had phosphorylation of Ser^376^ in exogenous MSK1s. However, MSK1 (D565A) and MSK1 (D195A/D565A), both of which lack C-terminal kinase activity, did not exhibit Ser^376^ phosphorylation in exogenous MSK1s (Fig. 3*B*). As expected, CREB phosphorylation by BMP-6 was suppressed in cells expressing MSK1 (D195A) and MSK1 (D195A/D565A) because CREB is a substrate for the N-terminal kinase in MSK1. Interestingly, CREB phosphorylation was still detected in the lysates of cells expressing MSK1 (D565A), which lacks C-terminal kinase activity. The phosphorylation of other amino acid residue(s) in MSK1 by BMP-6–activated p38 kinase may prompt direct activation of the N-terminal kinase in MSK1, in addition to its C-terminal kinase. To determine whether MSK1 mutants lose the ability to differentiate ATDC5 cells into chondrocytes, we cultured ATDC5 cells expressing WT MSK1 or its mutants at high density in the presence of BMP-6. All three MSK1 mutants exhibited lower chondrocyte differentiation ability than WT MSK1 (Fig. 3*C*). During chondrogenic differentiation induced by BMP stimulation, transcripts of the *Aggrecan* and *Col10a1* genes, both of which are ECM proteins produced by chondrocytes, are known to be induced (27, 28). BMP-6 increased the expression of aggrecan and Col10a1 mRNAs in parental ATDC5 cells, ATDC5 cells expressing WT MSK1, and ATDC5 cells expressing MSK1 (D195A/D565A) over time. However, overexpression of WT MSK1 in ATDC5 cells caused a greater increase in BMP-6–induced aggrecan and collagen10a1 mRNA expression than MSK1 (D195A/D565A) in ATDC5 cells. Nonetheless, equivalent mRNA expression was found between ATDC5 cells expressing MSK1 (D195A/D565A) and parental ATDC5 cells (Fig. 3, *D* and *E*). These results suggest that MSK1 (D195A/D565A) does not act as a dominant-negative mutant.

### Importance of amino acid residues phosphorylated by p38 kinase in MSK1

**Figure 4:**
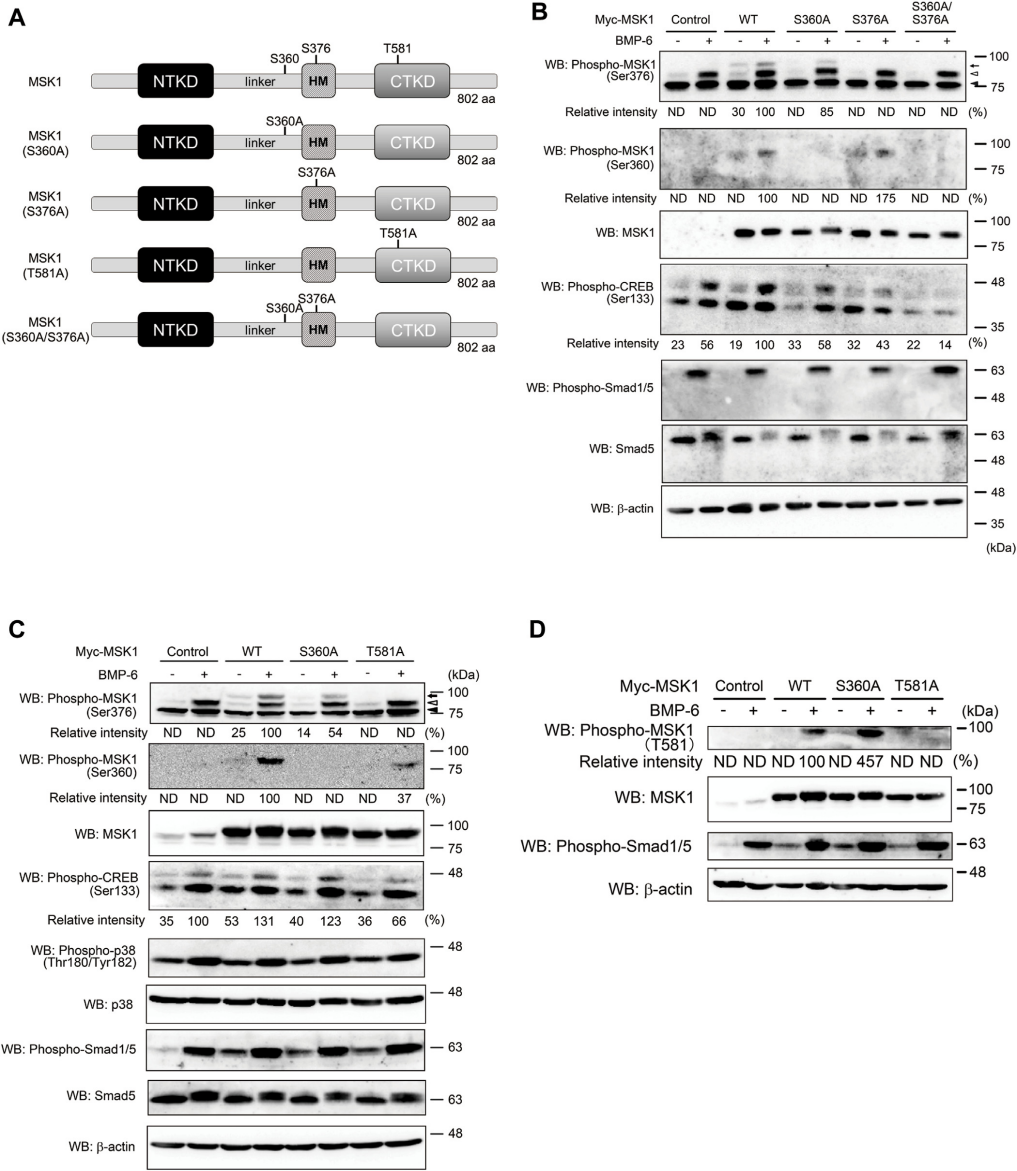

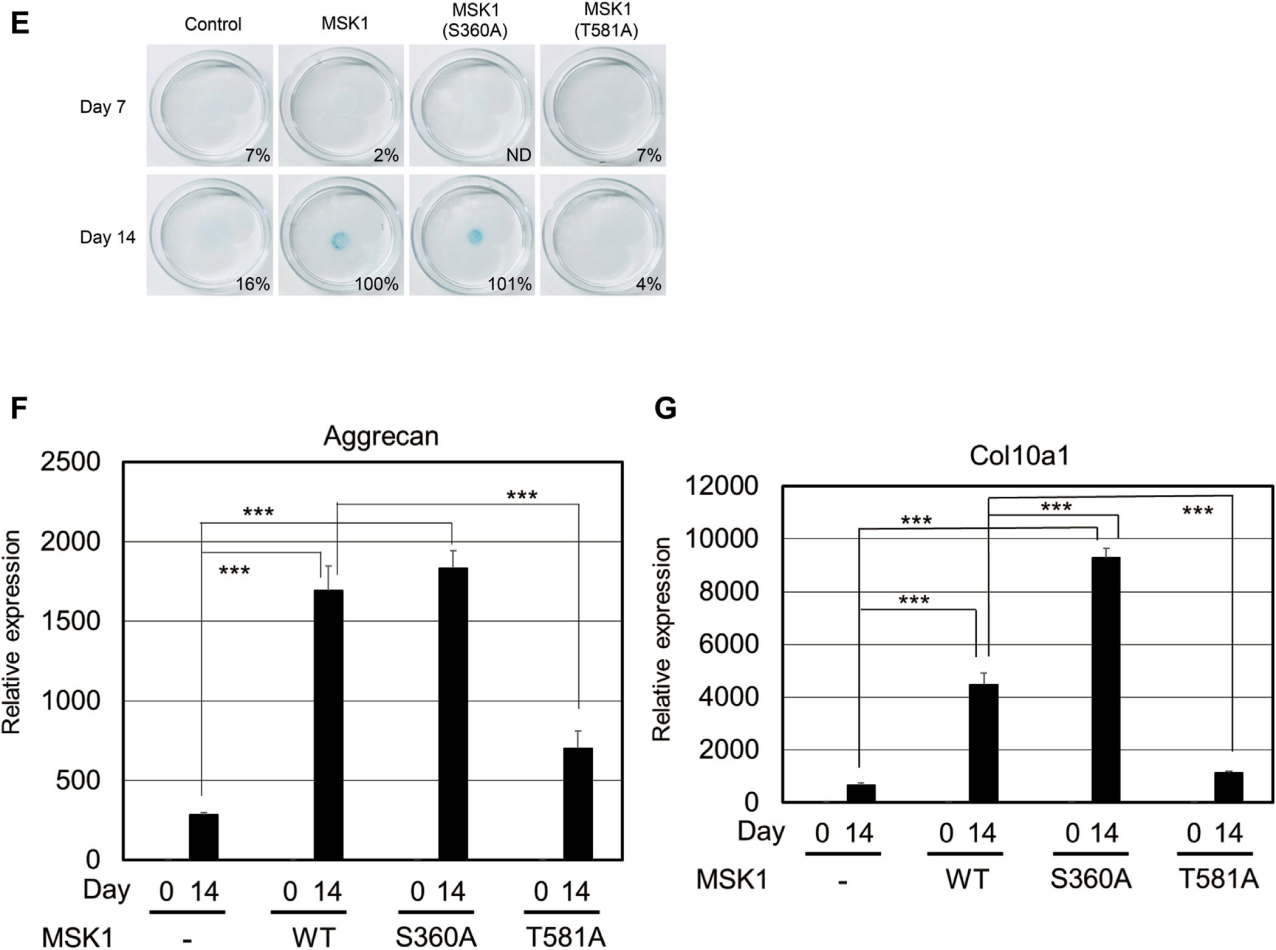
**MSK1 mutants lacking phosphorylation site targeted by p38 kinase and C-terminal kinase of MSK1.***A*, depiction of MSK1 and its mutants used in Figs. 4 and 5. *B*, phosphorylation of MSK1 or its mutants in ATDC5 cells stimulated with BMP-6. After expression of WT MSK1 or its mutants in ATDC5 cells using tetracycline as described in Fig. 3*B*, the cells were stimulated with 25 ng/ml BMP-6 for 1 h and their total lysates were prepared for Western blot analyses. The total expression levels of phospho-MSK1 (Ser376), phospho-MSK1 (Ser360), MSK1, phospho-CREB (Ser133), phospho-Smad1/5, Smad5, and β-actin are indicated in the *upper, second, third, fourth, fifth, sixth,* and *bottom panels*, respectively. A *black arrow* and a *white arrowhead* indicate ectopic and endogenous phospho-MSK1 (Ser376)-specific band, respectively. A *black arrowhead* indicates nonspecific bands. The intensity of the bands for phospho-MSK1 (Ser376) and phospho-MSK1 (Ser360) was normalized to the intensity of the band corresponding to MSK1. The intensity of the band for phospho-CREB (Ser133) was normalized to the intensity of the band corresponding to β-actin. Relative intensity was calculated with respect to the 1-h treatment of cells carrying WT MSK1 with BMP-6. *C*, comparison between serine-360 and threonine-581 in MSK1 as targeted amino acid residues for p38 kinase. After expression of WT MSK1 or its mutants in ATDC5 cells using tetracycline as described in Fig. 3*B*, the cells were stimulated with 25 ng/ml BMP-6 for 1 h and then their total lysates were prepared for Western blot analyses. The total expression levels of phospho-MSK1 (Ser376), phospho-MSK1 (Ser360), MSK1, phospho-CREB (Ser133), phospho-p38 (Thr180/Tyr182), p38, phospho-Smad1/5, Smad5, and β-actin are indicated in the *upper, second, third, fourth, fifth, sixth, seventh, eighth,* and *bottom panels*, respectively. A *black arrow* and a *white arrowhead* indicate ectopic and endogenous phospho-MSK1 (Ser376)-specific band, respectively. A *black arrowhead* indicates nonspecific bands. The intensity of the bands for phospho-MSK1 (Ser376) and phospho-MSK1 (Ser360) was normalized to the intensity of the band corresponding to MSK1. The intensity of the band for phospho-CREB (Ser133) was normalized to the intensity of the band corresponding to β-actin. Relative intensity was calculated with respect to the 1-h treatment of cells carrying WT MSK1 with BMP-6. *D*, phosphorylation of threonine at position 581 in MSK1 upon BMP-6 stimulation. As described in Fig. 3*B*, cell lysates were prepared from each transformant. The total expression levels of phospho-MSK1 (Thr581), MSK1, phospho-Smad1/5, and β-actin are indicated in the *upper, second, third,* and *bottom panels*, respectively. The intensity of the band for phospho-MSK1 (T581) was normalized to the intensity of the band corresponding to MSK1. Relative intensity was calculated with respect to the 1-h treatment of cells carrying WT MSK1 with BMP-6. *E*, chondrogenic differentiation of ATDC5 cells carrying WT MSK1 or its mutants in the presence of BMP-6. After the expression of WT MSK1 or its mutants in ATDC5 cells using tetracycline as described in Fig. 3*C*, the cells were reseeded at a high density and then stimulated with 25 ng/ml BMP-6. Seven (*upper panels*) or 14 days later (*lower panels*), the cells were stained with Alcian blue. Representative images are shown. The intensity of the area stained with Alcian blue was measured using ImageQuant TL. The relative intensity of the stained area was calculated with respect to the cells carrying WT MSK1 with BMP-6 alone for 14 days. *F* and *G*, expression of aggrecan and collagen 10a1 mRNAs in ATDC5 cells and their transformants. According to Fig. 3, *D* and *E*, ATDC5 cells expressing WT MSK1 or its mutants were cultured with 25 ng/ml BMP-6 for 14 days. Thereafter, total mRNAs from each transformant were prepared for qPCR to detect aggrecan (*F*) and collagen10a1 mRNAs (*G*). All values represent means ± SDs (n = 3). *Asterisks* indicate significant differences. BMP, bone morphogenetic protein; CREB, cAMP-response element-binding protein; MSK, mitogen- and stress-activated protein kinase; ND, not determined; qPCR, quantitative PCR.

The serine residue at position 360 in MSK1 is reported to be phosphorylated by p38 kinase. Thus, we prepared MSK1 (S360A) in addition to MSK1 (S376A) and MSK1 (S360A/S376A) because the C-terminal kinase in MSK1 can phosphorylate its serine residue at position 376 (Fig. 4*A*) (15, 16, 30). Each mutant was expressed in ATDC5 cells using the tet-on system. Indeed, BMP-6–mediated phosphorylation of Ser^360^ in MSK1 was not observed in ATDC5 cells expressing MSK1 (S360A) and MSK1 (S360A/S376A) (Fig. 4*B*). Furthermore, ATDC5 cells expressing MSK1 (S376A) and MSK1 (S360A/S376A) did not exhibit BMP-6–induced phosphorylation of Ser^376^ in MSK1. On the other hand, a decrease in Ser^376^ phosphorylation was observed in ATDC5 cells expressing MSK1 (S360A) upon BMP-6 stimulation. This result suggests that phosphorylation of Ser^360^ in MSK1 is crucial for activating the C-terminal kinase in MSK1. We hypothesized that p38 kinase may also be involved in the phosphorylation of other serine and/or threonine residues, excluding Ser^360^ in MSK1 (Fig. 4*B*). Consistent with the decreased phosphorylation of Ser^360^ and/or Ser^376^ in ATDC5 cells carrying mutant MSK1s following BMP-6 stimulation, CREB phosphorylation in these cells was weaker than that in cells expressing WT MSK1 (Fig. 4*B*).

In addition to Ser^360^ in MSK1, Thr^581^ in MSK1 is an amino acid residue targeted by p38 kinase (15, 16, 30). As the MSK1 (T581A) mutant possessed a serine residue at position 360, we could observe serine-360 phosphorylation of MSK1 (T581A) upon BMP-6 stimulation. However, BMP-6–mediated Ser^376^ phosphorylation could not be detected in ATDC5 cells carrying MSK1 (T581A), unlike in ATDC5 cells expressing WT MSK1. Consistent with the above results, CREB phosphorylation in ATDC5 cells expressing MSK1 (T581A) was markedly weaker than that in ATDC5 cells expressing WT MSK1 and MSK1 (S360A) upon BMP-6 stimulation (Fig. 4*C*). By investigating whether BMP-6–mediated phosphorylation of Thr^581^ in MSK1 could be detected in ATDC5 cells expressing MSK1 (T581A), we found that Thr^581^ phosphorylation of MSK1 does not occur in ATDC5 cells expressing MSK1 (T581A), although both WT MSK1 and MSK1 (S360A)-expressing ATDC5 cells exhibited BMP-6–induced phosphorylation of threonine-581 of MSK1 (Fig. 4*D*). Consistent with the phosphorylation of serine-376 of MSK1 in ATDC5 cells expressing WT MSK1 and MSK1 (S360A) upon BMP-6 stimulation, ATDC5 cells expressing WT MSK1 and MSK1 (S360A) differentiated into chondrocytes upon BMP-6 stimulation. However, BMP-6–induced chondrocyte differentiation did not occur in ATDC5 cells expressing MSK1 (T581A) (Fig. 4*E*). We also examined the mRNA expression of aggrecan and collagen10a1 mRNAs in these cells. As shown in Figure 4, *F* and *G*, aggrecan and collagen10a1 mRNAs were highly expressed in ATDC5 cells carrying WT MSK1 and MSK1 (S360A) upon stimulation with BMP-6. In ATDC5 cells expressing the MSK1 (T581A) mutant, the mRNA expression levels of aggrecan and collagen10a1 were markedly weaker 14 days after stimulation with BMP-6. These results suggest that phosphorylation of the 360th serine of MSK1 upon BMP-6 stimulation is necessary but insufficient for the differentiation of ATDC5 cells into chondrocytes. Conversely, the BMP-6–mediated phosphorylation of threonine-581 in MSK1 appears to be important for BMP-6–induced chondrocyte differentiation.

### Requirement of autophosphorylation of Ser^376^ in MSK1 by its C-terminal kinase for BMP-6–mediated chondrocyte differentiation

**Figure 5:**
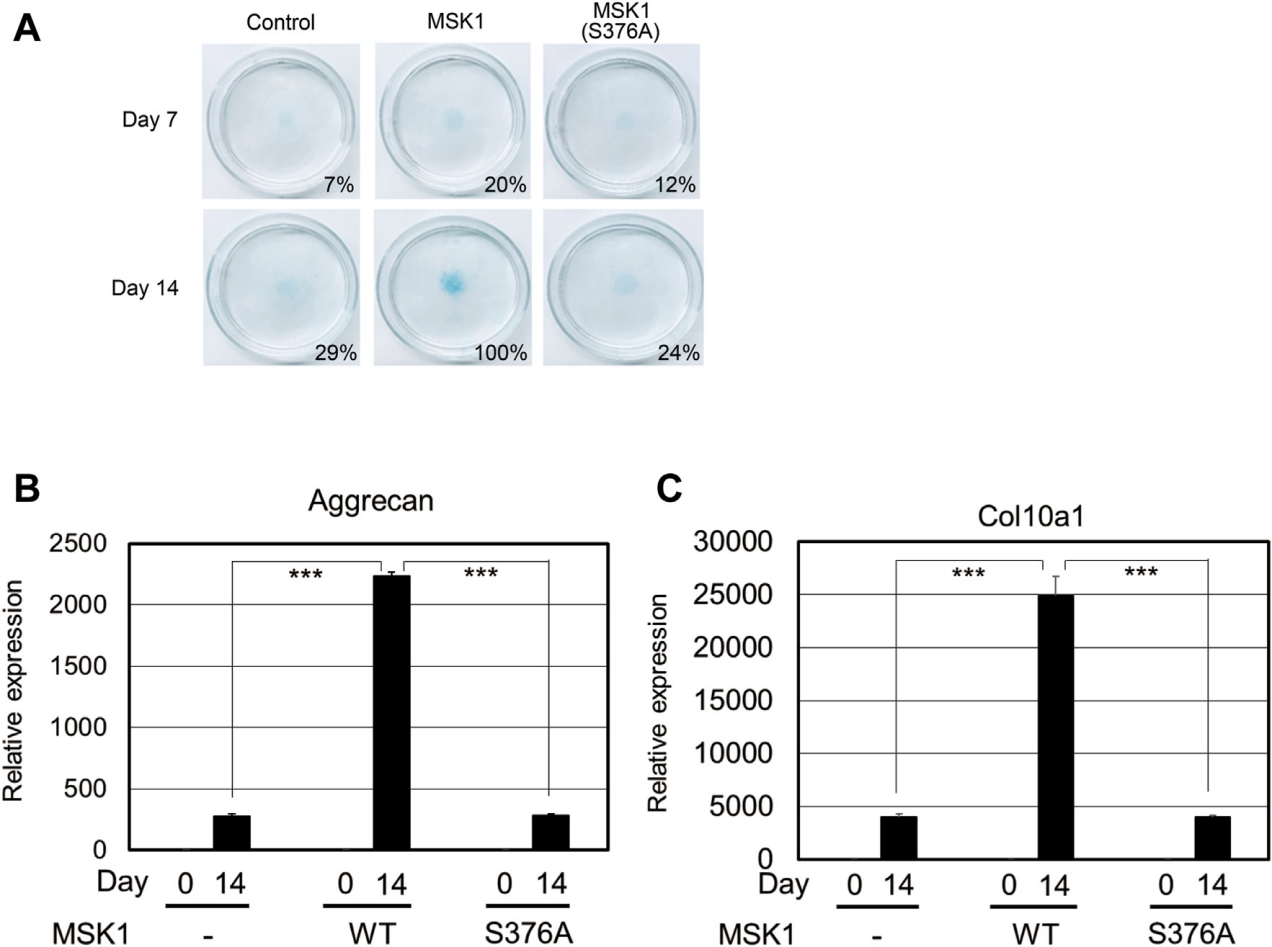
**Requirement of autophosphorylation of Ser**^**376**^**in MSK1 for its kinase activity upon BMP-6 stimulation.***A*, chondrogenic differentiation of ATDC5 cells carrying WT MSK1 or MSK1 (S376A) mutant in the presence of BMP-6. After the expression of WT MSK1 or MSK1 (S376A) mutant in ATDC5 cells using tetracycline as described in Fig. 3*C*, the cells were reseeded at a high density and then stimulated with 25 ng/ml BMP-6. Seven (*upper panels*) or 14 days later (*lower panels*), the cells were stained with Alcian blue. Representative images are shown. The intensity of the area stained with Alcian blue was measured using ImageQuant TL. The relative intensity of the stained area was calculated with respect to the cells carrying WT MSK1 with BMP-6 for 14 days. *B* and *C*, expression of aggrecan and collagen10a1 mRNAs in ATDC5 cells and their transformant. According to Fig. 3, *D* and *E*, ATDC5 cells expressing the WT MSK1 or its mutant were cultured with 25 ng/ml BMP-6 for 14 days. Thereafter, total mRNAs from each transformant were prepared for qPCR to detect aggrecan (*B*) and collagen10a1 mRNAs (*C*). All values represent means ± SDs (n = 3). *Asterisks* indicate significant differences. BMP, bone morphogenetic protein; MSK, mitogen- and stress-activated protein kinase; qPCR, quantitative PCR.

Ser^376^ in MSK1 is autophosphorylated by the C-terminal kinase in MSK1 (15, 16, 30). To examine whether phosphorylation of Ser^376^ in MSK1 is required for BMP-6–induced chondrocyte differentiation, we introduced the MSK1 (S376A) mutant into ATDC5 cells. As expected, Ser^376^ in MSK1 was not phosphorylated upon BMP-6 stimulation in ATDC5 cells carrying the MSK1 (S376A) mutant, despite the detection of BMP-6–induced phosphorylation of Smad1/5 in ATDC5 cells carrying the MSK1 (S376A) mutant. Consistent with these results, CREB phosphorylation was suppressed in ATDC5 cells carrying MSK1 (S376A) upon BMP-6 stimulation (Fig. 4*B*). When WT ATDC5 and its MSK1 (S376A) transformant were cultured in differentiation media containing BMP-6 for 14 days, MSK1 (S376A)–expressing ATDC5 cells marginally differentiated into chondrocytes, whereas ATDC5 cells expressing WT MSK1 fully differentiated (Fig. 5*A*). Consistent with the above results, MSK1 (S376A)–expressing ATDC5 cells exhibited induction of aggrecan and collagen10a1 mRNAs upon BMP-6 stimulation, similar to parental ATDC5 cells (Fig. 5, *B* and *C*).

### Effect of reduced expression of MSK1 on BMP-6–induced chondrocyte differentiation

To confirm that MSK1 is required for BMP-6–induced chondrocyte differentiation, we established ATDC5 cell lines carrying two shRNAs against MSK1 (shMSK1#2 and shMSK1#3) (Fig. 2*A*). When ATDC5 cells carrying shMSK1#2, shMSK1#3, or shControl were cultured with BMP-6 for 14 days, neither ATDC5 cells expressing shMSK1#2 nor shMSK1#3 differentiated into chondrocytes. However, ATDC5 cells expressing shControl were found to differentiate into chondrocytes (Fig. 2*B*).

### Involvement of the canonical Smad pathway in MSK1-induced chondrocyte differentiation

**Figure 6:**
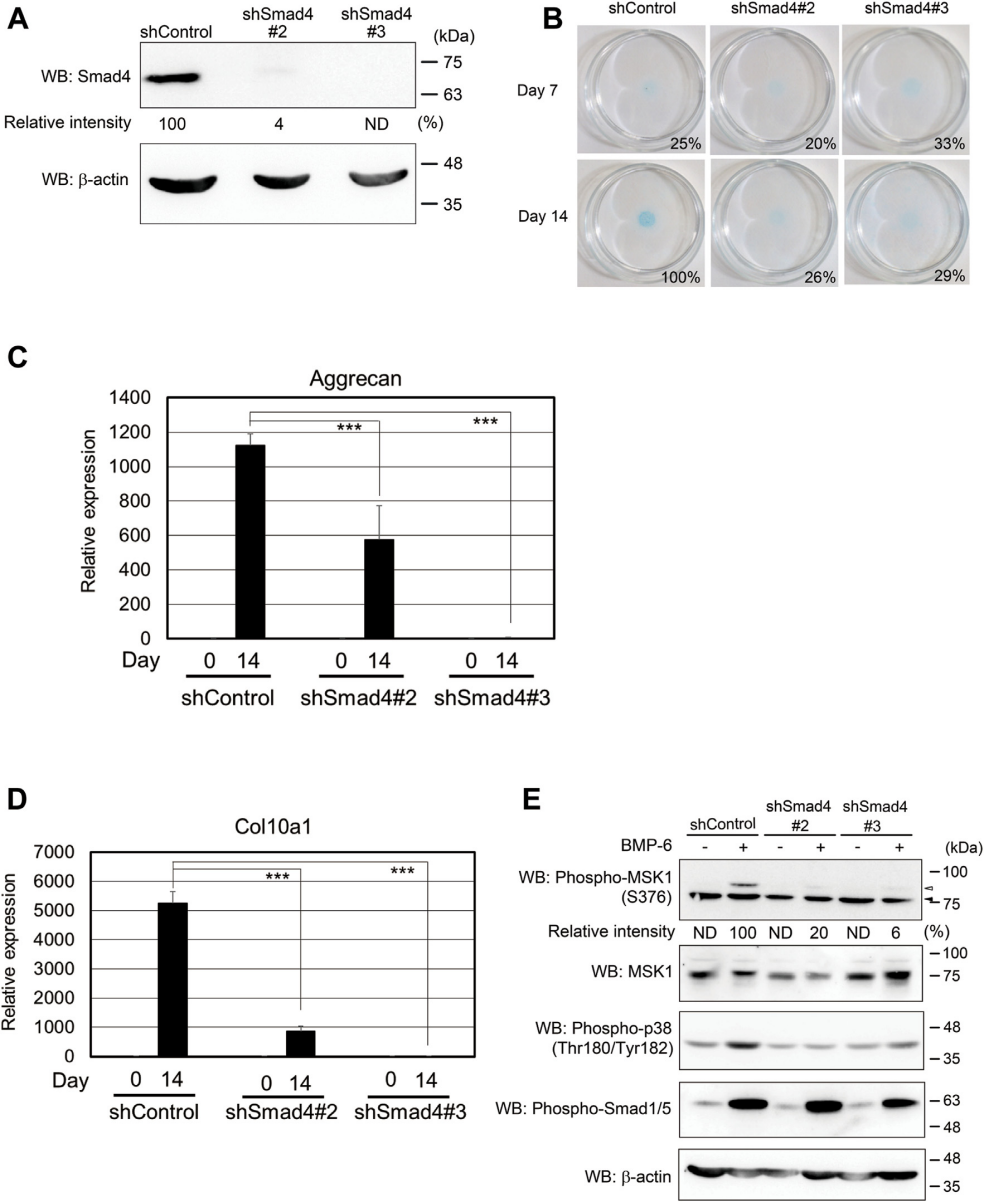
**Effect of the BMP/Smad-dependent pathway on BMP-6–mediated chondrogenic differentiation.***A*, expression of Smad4 in ATDC5 cells. Total lysates from each transformant were prepared for Western blot analyses. The total expression levels of Smad4 and β-actin are indicated in the *upper* and *lower panels*, respectively. The intensity of the band for Smad4 was normalized to the intensity of the band corresponding to β-actin. Relative intensity was calculated with respect to cells carrying shControl. *B*, chondrogenic differentiation of ATDC5 cells with decreased expression of Smad4 in the presence of BMP-6. The cells were seeded at a high density and then stimulated with 25 ng/ml BMP-6. Seven (*upper panels*) or 14 days later (*lower panels*), the cells were stained with Alcian blue. Representative images are shown. The intensity of the area stained with Alcian blue was measured using ImageQuant TL. The relative intensity of the stained area was calculated with respect to the cells carrying shControl with BMP-6 for 14 days. *C* and *D*, expression of aggrecan and collagen10a1 mRNA in ATDC5 cells with Smad4 knockdown. According to Fig. 2, *D* and *E*, ATDC5 cells with decreased expression of Smad4 were cultured with 25 ng/ml BMP-6 for 14 days. Thereafter, total mRNAs from each transformant were prepared for qPCR to detect aggrecan (*C*) and collagen10a1 mRNAs (*D*). All values represent means ± SDs (n = 3). *Asterisks* indicate significant differences. *E*, effect of Smad4 deficiency on BMP-6–induced p38 phosphorylation. The cells were stimulated with 25 ng/ml BMP-6 for 1 h and then their total lysates were prepared for Western blot analyses. The total expression levels of phospho-MSK1 (Ser376), MSK1, phospho-p38 (Thr180/Tyr182), phospho-Smad1/5, and β-actin are indicated in the *upper, second, third, fourth,* and *bottom panels*, respectively. The intensity of the band for phospho-MSK1 (S376) was normalized to the intensity of the band corresponding to MSK1. Relative intensity was calculated with respect to the 1-h treatment of cells carrying shControl with BMP-6. BMP, bone morphogenetic protein; MSK, mitogen- and stress-activated protein kinase; ND, not determined; qPCR, quantitative PCR.

**Figure 7:**
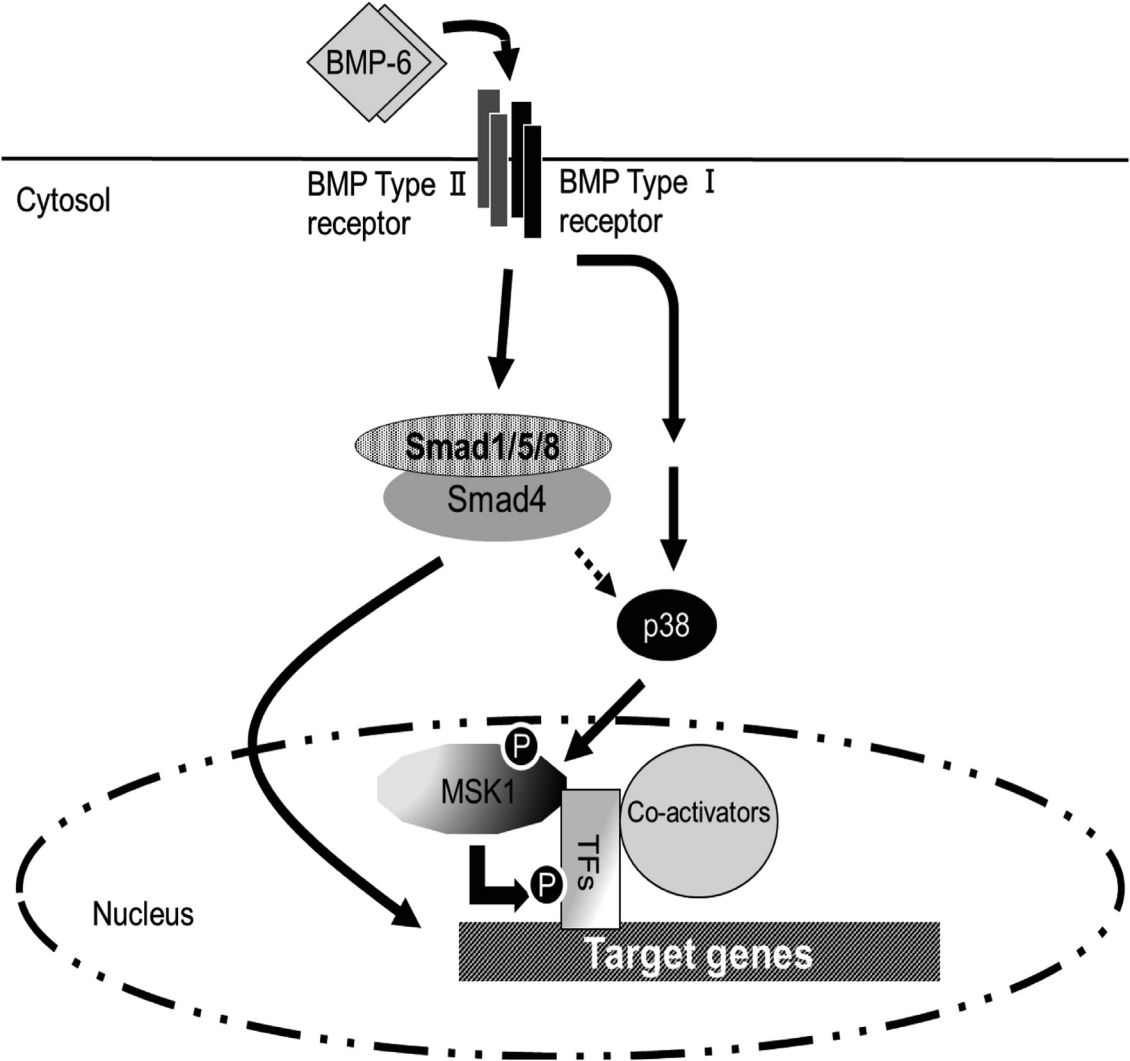
**Proposed model for BMP-6–mediated chondrocyte differentiation *via* synergy between the Smad-dependent and independent pathways.** After ATDC5 cells are stimulated with BMP-6, BMP receptors (BMP type I and type II receptors) activate Smad1/5, and p38 kinase. After p38 kinase phosphorylates MSK1, transcriptional factors, including CREB, are phosphorylated to possibly activate genes involved in chondrocyte differentiation. Furthermore, MSK1-mediated chondrocyte differentiation requires BMP-6/Smad signaling. BMP, bone morphogenetic protein; CREB, cAMP-response element-binding protein; MSK, mitogen- and stress-activated protein kinase.

We demonstrated that the canonical Smad pathway cooperates with noncanonical Smad pathway to fully activate TGF-β family signaling (2, 3). We knocked down Smad4 using two shRNAs corresponding to Smad4 (shSmad4#2 and shSmad4#3) (Fig. 6*A*) and stimulated the cells with BMP-6 for 7 or 14 days. As shown in Fig 6*B*, upon reduced expression of Smad4, the cells lost their ability to differentiate into chondrocytes following BMP-6 stimulation. Consistently, the BMP-6–induced expression of aggrecan and collagen10a1 mRNA was suppressed in ATDC5 cells carrying shSmad4#2 and shSmad4#3 (Fig. 6, *C* and *D*). Curiously, ATDC5 cells with reduced Smad4 expression exhibited a loss of BMP-6–mediated p38 phosphorylation, followed by decreased Ser^376^ phosphorylation of MSK1 (Fig. 6*E*). These results suggest that both the canonical and noncanonical Smad pathways are required for the differentiation of ATDC5 cells into chondrocytes in a BMP-6–dependent manner (Fig. 7).

## Discussion

Chondrocytes form cartilage together with several extracellular proteins including chondroitin sulfate, proteoglycan, and several collagens (6). Aggrecan is the most abundant proteoglycan in articular cartilage (4). Chondrocytes derived from MSCs play an important role not only in the maintenance of joints but also in skeletal development *via* endochondral ossification during embryonic development. The process of endochondral ossification is regulated by many secretary proteins, such as BMPs, FGFs, and IGFs, PTHrP, and Wnt (31).

BMPs have been implicated in ECM production, chondrocyte proliferation, and hypertrophy-like differentiation. When both *Smad1* and *Smad5* genes are specifically deleted in the cartilage, chondrodysplasia is observed (8). Thus, the canonical BMP signaling pathway appears to play a pivotal role in chondrocyte differentiation. However, the full activation of TGF-β family signaling requires both canonical and noncanonical pathways (2, 3).

As MSK1 is phosphorylated by the MAP kinase pathways including ERK, JNK, and p38 kinase (11), we speculated that BMPs involved in the activation of MAP kinases might activate MSK1 *via* MAP kinase pathways. Indeed, MSK1 was activated by p38 kinase, but not by ERK or JNK, in ATDC5 cells upon BMP stimulation. As shown in Fig. S1, osmotic stress induces JNK phosphorylation in ATDC5 cells, although JNK is not phosphorylated by BMP-6 in ATDC5 cells. Thus, endogenous JNK protein is expressed in ATDC5 cells. Consistently, BMP-6–mediated differentiation of ATDC5 cells into chondrocytes was inhibited by a p38 kinase inhibitor, but not by an MEK inhibitor.

To confirm that MSK1 pathway activated upon BMP-6 stimulation is required in cell line other than ATDC-5, we stimulated C2C12 cells (a myoblast cell line), HaCaT cells (a keratinocyte cell line) and MCF10A1 cells (breast epithelial cell line) with BMP-6 for 1 h. As shown in Fig. S3*A*, the BMP-6–mediated phosphorylation of Ser^376^ in MSK1 was detected in C2C12 cells, but not in HaCaT nor MCF10A1 cells. C2C12 cells are known to differentiate into osteoblasts upon BMP stimulation (26, 32). As demonstrated in Fig. S3*B*, BMP-6 promoted the osteoblastic differentiation in C2C12 cells, which was suppressed in the presence of dorsomorphin, SB203580, and SB747651A, but not U0126. Thus, these findings suggest that similar to ATDC5 cells, C2C12 cells differentiate into osteoblasts through the phosphorylation of MSK1 by p38 kinase in response to BMP-6 stimulation.

The C-terminal kinase in MSK1 has been reported to prompt autophosphorylation of MSK1 to activate its N-terminal kinase (12, 15, 16, 30). However, our experiments using N-terminal and/or C-terminal kinase–defective mutants (MSK1 (D195A), MSK1 (D565A), and MSK1 (D195A/D565A)) revealed BMP-6–induced differentiation of ATDC5 cells into chondrocytes to some extent, despite their weaker abilities than WT MSK1. Thus, these three mutants could not become dominant-negative mutants for endogenous MSK1, despite the loss or reduction of their kinase activities (Fig. 3*B*).

p38 kinase has been reported to phosphorylate Ser^360^ and Thr^581^ in MSK1. Furthermore, phosphorylation of these amino acids seems to play an essential role in the C-terminal kinase activity of MSK1 (11, 15, 16, 30). Based on our results, BMP-6–mediated activation of MSK1 in ATDC5 cells may require phosphorylation of Thr^581^, but not Ser^360^.

The C-terminal kinase in MSK1 targets Ser^212^, Ser^376^, and Ser^381^ in MSK1. Of the three phosphorylated amino acid residues, Ser^376^ in MSK1 is essential for the activation of its N-terminal kinase (15, 16, 30). Therefore, we substituted the serine residue at position 376 with alanine in MSK1. This single amino acid substitution perturbed BMP-6–meidated differentiation of ATDC5 cells into chondrocytes and BMP-6–induced expression of ECM proteins (Fig. 5, *A*–*C*). Taken together, these results suggest that activation of N-terminal kinase in MSK1 *via* BMP-6 signaling promotes the differentiation of ATDC5 cells into chondrocytes.

To prove that MSK1 is involved in BMP-6–induced chondrogenic differentiation, a loss-of-function study of MSK1 was performed. As expected, two independent short hairpin RNAs targeting MSK1 completely blocked BMP-6–induced chondrogenic differentiation. Thus, the BMP-6/p38/MSK1 axis may be involved in the differentiation into chondrocytes when ATDC5 cells are stimulated with BMP-6.

The TGF-β family transmits intracellular signals through Smad-dependent and Smad-independent pathways, known as canonical and noncanonical TGF-β family signaling, respectively. In most cases, full activation of the TGF-β family signaling requires the engagement of both the canonical and noncanonical Smad signaling pathways. Unexpectedly, knockdown of Smad4 suppressed BMP-6–induced phosphorylation of MSK1. According to several studies, p38 kinase is activated by GADD45β (33, 34). In particular, GADD45β is induced *via* the Smad pathway to interact with mitogen-activated protein kinase kinase kinase 4 (MEKK4 or MTK1), which activates p38 kinase (35, 36, 37, 38). The overexpression of GADD45β alone in ATDC5 cells with decreased Smad4 expression (shSmad4#2 and shSmad4#3) was found to result in the recovery of chondrogenic differentiation, even in the absence of BMP-6 (Fig. S4, *A* and *B*). These results suggest that GADD45β may lead to the differentiation of ATDC5 cells into chondrocytes by enhancing the phosphorylation of MSK1.

As van der Heide *et al.* demonstrated that the TGF-β/Smad pathway induces GADD45β to enhance the activity of MSK1 *via* p38 kinase (24), we investigated whether phosphorylation of Ser^376^ in MSK1 could be induced in ATDC5 cells upon TGF-β stimulation, such as BMP-6 stimulation. As shown in Fig. S5*A*, Ser^376^ in MSK1 was phosphorylated when cells were stimulated with TGF-β. However, the expression of aggrecan and collagen10a1 mRNAs was not induced by TGF-β, unlike BMP-6 (Fig. S5, *B* and *C*). Thus, besides MSK1 activation, BMP-6–mediated differentiation of ATDC5 cells into chondrocytes requires the BMP-6–mediated pathway(s).

Several studies have reported on the complete knockout (KO) of MSK1 and its family member MSK2 in mice. However, no studies have specifically investigated chondrocyte differentiation in MSK1 and/or MSK2 KO mice. To date, MSK1 KO mice have not exhibited any noticeable phenotypes. In contrast, double KO mice for MSK1 and MSK2 have shown hypersensitivity to lipopolysaccharide-induced endotoxic shock and prolonged inflammation as well as reduced neurite arborization (11, 22, 39, 40, 41, 42, 43). We hypothesize that chondrocyte-specific knockout of MSK1 could potentially lead to defects in chondrocyte differentiation.

## Experimental procedures

### Antibodies

The antibodies used are listed in Table S1.

### Reagents

U0126 (cat. no. 211-01051), SB203580 (cat. no. 195-16553) and dorsomorphin (cat. no. 041-31223) were purchased from FUJIFILM Wako Pure Chemical Corporation. SB747651A (cat. no. 4630) was obtained from Tocris.

### Cell culture

Mouse ATDC5 cells (RCB0565) (44) purchased from RIKEN BRC were cultured in Dulbecco's Modified Eagle Medium (DMEM)/F-12 (1:1 mixture of DMEM (Nacalai tesque) and Ham's Nutrient Mixture F-12 (Ham's F-12; Nacalai tesque)) containing 5% fetal calf serum (FCS; Biological Industries) and penicillin/streptomycin solution (PS, Nacalai tesque), termed DMEM/F-12/5% FCS/PS. MCF10A1 cells were cultured in DMEM/F-12/5% FCS/5% horse serum (cat. H1138, Sigma-Aldrich)/PS supplemented with 10 mM Hepes (pH 7.5), 10 μg/ml insulin (cat. 093-06351, FUJIFILM Wako Pure Chemical Corporation), 20 ng/ml EGF (cat. 058-09521, FUJIFILM Wako Pure Chemical Corporation), 0.5 μg/ml hydrocortisone (cat. 086-10191, FUJIFILM Wako Pure Chemical Corporation), and 5 mM folskolin (cat. 067-02191, FUJIFILM Wako Pure Chemical Corporation). 293T, C2C12, and HaCaT cells were cultured in DMEM/10% FCS/PS.

### Micoromass cell culture

A total of 2.5 × 10^7^ cells/ml ATDC5 cells were suspended in DMEM/F-12/5% FCS/PS. Ten microliters of this suspension was spotted in the center of a 3.5 cm dish and incubated at 37 °C and 5% CO_2_ for 1 h to allow cells to settle. The cells were then incubated in 1 ml of DMEM/F-12/5% FCS/PS for 16 h. If necessary, DMEM/F-12/5% FCS/PS supplemented with or without 25 ng/ml BMP-6 (cat. no. 120-06, PeproTech) was changed every other day, and the cells were cultured for the indicated days. After removing the medium from cells using the micromass cell culture method, the cells were washed twice with 1× PBS and fixed in 3.7% formalin solution for 10 min. Subsequently, the cells were washed twice with 1× PBS, and then three times every 3 min with 0.1 M HCl. The cells were stained with 0.25% Alcian blue solution (cat. no. A5268, Sigma-Aldrich) in 0.1 M HCl for 30 min at room temperature. After three washes with 0.1 M HCl every 3 min, the cells were briefly rinsed with tap water for a few seconds and air-dried at room temperature.

### Culture for mouse embryonic metatarsals

The second, third, and fourth metatarsal bone rudiments were harvested from C57BL/6 mouse embryos at 17.5 days postmating. Subsequently, the bones were cultured in DMEM/F-12 medium supplemented with 10% FCS and PS, as previously described (28, 45). The cultured bones were fixed in 96% ethanol for 1 h and then stained with 0.25% Alcian blue solution in 0.1 M HCl for 6 h at room temperature. Following staining, the bones were dehydrated overnight in ethanol. The dehydrated bones were immersed in 1% KOH for 1 h, followed by staining with 0.001% Alizarin Red S (cat. 013-25452, FUJIFILM Wako Pure Chemical Corporation) in 1% KOH overnight. After staining, the bones were sequentially washed with 25% glycerol for 10 min, 50% glycerol for 10 min, and 80% glycerol for 10 min. Finally, the stained bones were stored in glycerol. Images were captured using an inverted microscope (TS100, Nikon). Animal work was performed with institutional approval by the Animal Care and Use Committee of Showa Pharmaceutical University (approval P-2024-5, dated April 15, 2024).

### RNA preparation and quantitative PCR analysis

Total RNAs from ATDC5 cells and their transformants were extracted using a NucleoSpin RNA (cat. no. 740955, Macherey-Nagel, Düren). Reverse transcription was performed using a High-Capacity RNA-to-complementary deoxyribonucleic acid (cDNA) kit (cat. No. 4387406, Thermo Fisher Scientific). Quantitative PCR (qPCR) was performed using a KAPA SYBR Fast qPCR kit (cat. No. KK4600, KAPA Biosystems). All reactions were carried out using a Thermal Cycler Dice (TAKARA). Each sample was analyzed at least twice in triplicate for each PCR measurement. Melting curves were checked to ensure specificity. Relative quantification of mRNA expression was calculated using the standard curve method with the β-actin level. Prior to qPCR, agarose gel electrophoresis was performed to confirm that the DNA fragment amplified using each primer set was a single band of the correct size. The primer sets are listed in Table S2.

### Western blot analysis

ATDC5 cells and their transformants were lysed using 500 μl of TNE buffer (10 mM Tris [pH 7.4], 150 mM NaCl, 1 mM EDTA, 1% NP-40, 1 mM PMSF, 5 μg/ml leupeptin, 100 U/ml aprotinin, 2 mM sodium vanadate, 40 mM NaF, and 20 mM β-glycerophosphate). Cell lysates were boiled for 5 min in sample buffer, separated by SDS-PAGE, and transferred onto UltraCruz Nitrocellulose Pure Transfer membranes (Santa Cruz Biotechnology). The membranes were then probed with the indicated primary antibodies, which were detected using horseradish peroxidase–conjugated secondary antibodies and a chemiluminescent substrate (cat. no. T7101A, Western BLoT Quant HRP Substrate, TAKARA). The intensity of each band was measured using ImageQuant TL analysis software (Cytiva) after each gel image was captured with the ImageQuant LAS 4000 mini (Amersham).

### Expression plasmids

Human MSK1 and GADD45β were cloned from human HaCaT cell–derived cDNAs. N-terminal kinase–defective MSK1 (D195A), C-terminal kinase–defective MSK1 (D565A), N- and C-terminal kinase–defective MSK1 (D195A/D565A), MSK1 (S360A), MSK1 (T581A), and MSK1 (S376A) (15, 16, 30) were prepared using PrimeStar HS DNA Polymerase (cat. no. R010A, TAKARA). After confirming their nucleotide sequences, the fragments were inserted into the pcDEF3 vector with an Myc tag at the N terminus (46). All constructs were subcloned into pENTER4 (cat. no. A10561, Thermo Fisher Scientific). Using the Gateway LR Clonase II enzyme mix (cat. no. 11791020, Thermo Fisher Scientific), Myc-MSK1 cDNA cloned into pENTER4 was further integrated into pInducer20 (cat. no. #44012, Addgene), one of the tet-on expression vectors, to create the lentiviral expression vector for Myc-MSK1 and its mutants. Each lentiviral vector (4 μg) was transfected into 293T cells (5 × 10^6^ cells/10 cm dish) with VSV (1.4 μg), GAG (2.6 μg), and REV (2.0 μg) in 500 μl of DMEM containing 100 μg polyethylenimine linear (MW 25000) (cat. no. 23966, Polysciences). After 48 h of transfection, the medium was collected as the lentiviral source. Each lentivirus was incubated in DMEM containing 8 μg/ml polybrene (cat. no. H9268, Sigma-Aldrich) for 2 h and then added to ATDC5 cell culture dishes. Twelve hours after infection, the cells were washed and cultured in medium containing 1 mg/ml G-418 sulfate (cat. no. 076-05962, FUJIFILM Wako Pure Chemical Corporation). Infected ATDC5 cells that became G418-resistant were used in the experiments. When the tet-on system was in operation, tetracycline hydrochloride (1.5–2.0 mg/ml, cat. no. 209-16561, FUJIFILM Wako Pure Chemical Corporation) was added to the medium 16 h before the experiments.

### Lentiviral shRNAs for MSK1 and Smad4

The lentiviral vectors for MSK1 and Smad4 shRNAs were constructed using a pLKO.1-TRC vector (cat. no. 10878, Addgene) (47). The sequences for shMSK1#2 and shMSK1#3 are 5′- CCGGAGACCTACTTCAGCGTCTCTTctcgagAAGAGACGCTGAAGTAGGTCTTTTTG-3′/3′- TCTGGATGAAGTCGCAGAGAAgagctcTTCTCTGCGACTTCATCCAGAAAAAACTTAA-5′ and 5′- CCGGCAGAAGAAATCAAAGAACATCTCTTctcgagAAGAGATGTTCTTTGATTTCTTCTGT-3′/3′- TCTTCTTTAGTTTCTTGTAGAGAAgagctcTTCTCTACAAGAAACTAAAGAAGACAAAAACTTAA-5′, respectively. The sequences for shSmad4#2 and shSmad4#3 are 5′- CCGGGGAATTGATCTCTCAGGATTActcgagTAATCCTGAGAGATCAATTCCTTTTTG-3′/3′- CCTTAACTAGAGAGTCCTAATgagctcATTAGGACTCTCTAGTTAAGGAAAAACTTAA -5′ and 5′- CCGGGCCATAGTGAAGGACTGTTGCctcgagGCAACAGTCCTTCACTATGGCTTTTTG-3′/3′- CGGTATCACTTCCTGACAACGgagctcCGTTGTCAGGAAGTGATACCGAAAAACTTAA-5′, respectively. Each lentiviral vector (4 μg) was transfected into 293T cells (5 × 10^6^ cells/10 cm dish) with psPAX2 (3.5 μg) and pMD2.G (2.5 μg), according to the method for constructing the lentivirus expression vector. After 48 h of infection, the medium was collected as the lentiviral source. Each lentivirus was incubated in DMEM containing 8 μg/ml polybrene for 2 h and then added to ATDC5 cell culture dishes. Twelve hours after infection, the cells were washed and cultured in medium containing 5 μg/ml puromycin dihydrochloride (cat. no. 164-23154, FUJIFILM Wako Pure Chemical Corporation). Infected ATDC5 cells that became puromycin-resistant were used in the experiments.

### Statistical analysis

All statistical analyses were performed using EZR version 1.52 (Saitama Medical Center, Jichi Medical University), which is a graphical user interface for R version 4.02 (The R Foundation for Statistical Computing) (48). Significance was assessed using one-way ANOVA. Probability values below 0.05, 0.01, and 0.001 were considered significant; ∗*p* < 0.05, ∗∗*p* < 0.01, and ∗∗∗*p* < 0.001.

## Data availability

All data generated during this study are included in this article. The data that are not described in the current manuscript can be obtained from the corresponding author (Susumu Itoh, sitoh@ac.shoyaku.ac.jp) upon request.

## Supporting information

This article contains supporting information (26, 32, 49, 50, 51, 52, 53).

## Conflict of interest

The authors declare that they have no conflicts of interest with the contents of this article.

## References

[1] Katagiri T., Watabe T. (2016). Bone morphogenetic proteins. Cold Spring Harbor Perspect. Biol..

[2] Itoh S., Thorikay M., Kowanetz M., Moustakas A., Itoh F., Heldin C.H. (2003). Elucidation of Smad requirement in transforming growth factor-β type I receptor-induced responses. J. Biol. Chem..

[3] Zhang Y.E. (2017). Non-Smad signaling pathways of the TGF-β family. Cold Spring Harbor Perspect. Biol..

[4] Thielen N.G.M., van der Kraan P.M., van Caam A.P.M. (2019). TGFβ/BMP signaling pathway in cartilage homeostasis. Cells.

[5] Zuscik M.J., Hilton M.J., Zhang X., Chen D., O'Keefe R.J. (2008). Regulation of chondrogenesis and chondrocyte differentiation by stress. J. Clin. Invest..

[6] Usami Y., Gunawardena A.T., Iwamoto M., Enomoto-Iwamoto M. (2016). Wnt signaling in cartilage development and diseases: lessons from animal studies. Lab. Invest..

[7] Chen H., Tan X.N., Hu S., Liu R.Q., Peng L.H., Li Y.M. (2021). Molecular mechanisms of chondrocyte proliferation and differentiation. Front. Cell Dev. Biol..

[8] Retting K.N., Song B., Yoon B.S., Lyons K.M. (2009). BMP canonical Smad signaling through Smad1 and Smad5 is required for endochondral bone formation. Development.

[9] Wang M., Jin H., Tang D., Huang S., Zuscik M.J., Chen D. (2011). Smad1 plays an essential role in bone development and postnatal bone formation. Osteoarthritis Cartilage.

[10] Adewumi I., Lopez C., Davie J.R. (2019). Mitogen and stress- activated protein kinase regulated gene expression in cancer cells. Adv. Biol. Regul..

[11] Sattarifard H., Safaei A., Khazeeva E., Rastegar M., Davie J.R. (2023). Mitogen- and stress-activated protein kinase (MSK1/2) regulated gene expression in normal and disease states. Biochem. Cel. Biol..

[12] Deak M., Clifton A.D., Lucocq L.M., Alessi D.R. (1998). Mitogen- and stress-activated protein kinase-1 (MSK1) is directly activated by MAPK and SAPK2/p38, and may mediate activation of CREB. EMBO J..

[13] Arencibia J.M., Pastor-Flores D., Bauer A.F., Schulze J.O., Biondi R.M. (2013). AGC protein kinases: from structural mechanism of regulation to allosteric drug development for the treatment of human diseases. Biochim. Biophys. Acta.

[14] Caenepeel S., Charydczak G., Sudarsanam S., Hunter T., Manning G. (2004). The mouse kinome: discovery and comparative genomics of all mouse protein kinases. Proc. Natl. Acad. Sci. U. S. A..

[15] McCoy C.E., Campbell D.G., Deak M., Bloomberg G.B., Arthur J.S. (2005). MSK1 activity is controlled by multiple phosphorylation sites. Biochem. J..

[16] Arthur J.S. (2008). MSK activation and physiological roles. Front. Biosci..

[17] Vermeulen L., Vanden Berghe W., Beck I.M., De Bosscher K., Haegeman G. (2009). The versatile role of MSKs in transcriptional regulation. Trends Biochem. Sci..

[18] Park J., Al-Ramahi I., Tan Q., Mollema N., Diaz-Garcia J.R., Gallego-Flores T. (2013). RAS-MAPK-MSK1 pathway modulates ataxin 1 protein levels and toxicity in SCA1. Nature.

[19] Cheng M.B., Zhang Y., Cao C.Y., Zhang W.L., Zhang Y., Shen Y.F. (2014). Specific phosphorylation of histone demethylase KDM3A determines target gene expression in response to heat shock. PLoS Biol..

[20] Chakraborty A., Diefenbacher M.E., Mylona A., Kassel O., Behrens A. (2015). The E3 ubiquitin ligase Trim7 mediates c-Jun/AP-1 activation by Ras signalling. Nat. Commun..

[21] Singh K., Cassano M., Planet E., Sebastian S., Jang S.M., Sohi G. (2015). A KAP1 phosphorylation switch controls MyoD function during skeletal muscle differentiation. Genes Dev..

[22] Reyskens K.M., Arthur J.S. (2016). Emerging roles of the mitogen and stress activated kinases MSK1 and MSK2. Front. Cell. Dev. Biol..

[23] Abecassis L., Rogier E., Vazquez A., Atfi A., Bourgeade M.F. (2004). Evidence for a role of MSK1 in transforming growth factor-β-mediated responses through p38α and Smad signaling pathways. J. Biol. Chem..

[24] van der Heide L.P., van Dinther M., Moustakas A., ten Dijke P. (2011). TGFβ activates mitogen- and stress-activated protein kinase-1 (MSK1) to attenuate cell death. J. Biol. Chem..

[25] Wu M., Chen G., Li Y.P. (2016). TGF-β and BMP signaling in osteoblast, skeletal development, and bone formation, homeostasis and disease. Bone Res..

[26] Fujii M., Takeda K., Imamura T., Aoki H., Sampath T.K., Enomoto S. (1999). Roles of bone morphogenetic protein type I receptors and Smad proteins in osteoblast and chondroblast differentiation. Mol. Biol. Cell.

[27] Kawamura I., Maeda S., Imamura K., Setoguchi T., Yokouchi M., Ishidou Y. (2012). SnoN suppresses maturation of chondrocytes by mediating signal cross-talk between transforming growth factor-β and bone morphogenetic protein pathways. J. Biol. Chem..

[28] Kakoi H., Maeda S., Shinohara N., Matsuyama K., Imamura K., Kawamura I. (2014). Bone morphogenic protein (BMP) signaling up-regulates neutral sphingomyelinase 2 to suppress chondrocyte maturation via the Akt protein signaling pathway as a negative feedback mechanism. J. Biol. Chem..

[29] Alvarez J., Sohn P., Zeng X., Doetschman T., Robbins D.J., Serra R. (2002). TGFβ2 mediates the effects of hedgehog on hypertrophic differentiation and PTHrP expression. Development.

[30] McCoy C.E., macdonald A., Morrice N.A., Campbell D.G., Deak M., Toth R. (2007). Identification of novel phosphorylation sites in MSK1 by precursor ion scanning MS. Biochem. J..

[31] Kronenberg H.M. (2003). Developmental regulation of the growth plate. Nature.

[32] Katagiri T., Yamaguchi A., Komaki M., Abe E., Takahashi N., Ikeda T. (1994). Bone morphogenetic protein-2 converts the differentiation pathway of C2C12 myoblasts into the osteoblast lineage. J. Cel. Biol..

[33] Salvador J.M., Brown-Clay J.D., Fornace A.J. (2013). Gadd45 in stress signaling, cell cycle control, and apoptosis. Adv. Exp. Med. Biol..

[34] Humayun A., Fornace A.J. (2022). GADD45 in stress signaling, cell cycle control, and apoptosis. Adv. Exp. Med. Biol..

[35] Takekawa M., Tatebayashi K., Itoh F., Adachi M., Imai K., Saito H. (2002). Smad-dependent GADD45β expression mediates delayed activation of p38 MAP kinase by TGF-β. EMBO J..

[36] Yoo J., Ghiassi M., Jirmanova L., Balliet A.G., Hoffman B., Fornace A.J. (2003). Transforming growth factor-β-induced apoptosis is mediated by Smad-dependent expression of GADD45β through p38 activation. J. Biol. Chem..

[37] Major M.B., Jones D.A. (2004). Identification of a gadd45β 3' enhancer that mediates SMAD3- and SMAD4-dependent transcriptional induction by transforming growth factor β. J. Biol. Chem..

[38] Ungefroren H., Groth S., Ruhnke M., Kalthoff H., Fandrich F. (2005). Transforming growth factor-β (TGF-β) type I receptor/ALK5-dependent activation of the GADD45β gene mediates the induction of biglycan expression by TGF-β. J. Biol. Chem..

[39] Wiggin G.R., Soloaga A., Foster J.M., Murray-Tait V., Cohen P., Arthur J.S. (2002). MSK1 and MSK2 are required for the mitogen- and stress-induced phosphorylation of CREB and ATF1 in fibroblasts. Mol. Cell. Biol..

[40] Brami-Cherrier K., Valjent E., Herve D., Darragh J., Corvol J.C., Pages C. (2005). Parsing molecular and behavioral effects of cocaine in mitogen- and stress-activated protein kinase-1-deficient mice. J. Neurosci..

[41] Ananieva O., Darragh J., Johansen C., Carr J.M., McIlrath J., Park J.M. (2008). The kinases MSK1 and MSK2 act as negative regulators of Toll-like receptor signaling. Nat. Immunol..

[42] Chang S., Iversen L., Kragballe K., Arthur J.S., Johansen C. (2011). Mice lacking MSK1 and MSK2 show reduced skin tumor development in a two-stage chemical carcinogenesis model. Cancer Invest..

[43] McGuire V.A., Rosner D., Ananieva O., Ross E.A., Elcombe S.E., Naqvi S. (2017). β interferon production is regulated by p38 mitogen-activated protein kinase in macrophages via both MSK1/2- and tristetraprolin-dependent pathways. Mol. Cell. Biol..

[44] Atsumi T., Miwa Y., Kimata K., Ikawa Y. (1990). A chondrogenic cell line derived from a differentiating culture of AT805 teratocarcinoma cells. Cell Differ. Dev..

[45] Houston D.A., Staines K.A., MacRae V.E., Farquharson C. (2016). Culture of murine embryonic metatarsals: a physiological model of endochondral ossification. J. Vis. Exp..

[46] Goldman L.A., Cutrone E.C., Kotenko S.V., Krause C.D., Langer J.A. (1996). Modifications of vectors pEF-BOS, pcDNA1 and pcDNA3 result in improved convenience and expression. Biotechniques.

[47] Moffat J., Grueneberg D.A., Yang X., Kim S.Y., Kloepfer A.M., Hinkle G. (2006). A lentiviral RNAi library for human and mouse genes applied to an arrayed viral high-content screen. Cell.

[48] Kanda Y. (2013). Investigation of the freely available easy-to-use software 'EZR' for medical statistics. Bone Marrow Transpl..

[49] Persson U., Izumi H., Souchelnytskyi S., Itoh S., Grimsby S., Engström U. (1998). The L45 loop in type I receptors for TGF-β family members is a critical determinant in specifying Smad isoform activation. FEBS Lett..

[50] Nakao A., Imamura T., Souchelnytskyi S., Kawabata M., Ishisaki A., Oeda E. (1997). TGF-β receptor-mediated signalling through Smad2, Smad3 and Smad4. EMBO J..

[51] Tamaki K., Souchelnytskyi S., Itoh S., Nakao A., Sampath K., Heldin C.H. (1998). Intracellular signaling of osteogenic protein-1 through Smad5 activation. J. Cell. Physiol..

[52] Zhou F., Zhang L., van Laar T., van Dam H., ten Dijke P. (2011). GSK3β inactivation induces apoptosis of leukemia cells by repressing the function of c-Myb. Mol. Biol. Cell.

[53] Ikeno S., Nakano N., Sano K., Minowa T., Sato W., Akatsu R. (2019). PDZK1-interacting protein 1 (PDZK1IP1) traps Smad4 protein and suppresses transforming growth factor-β (TGF-β) signaling. J. Biol. Chem..

