# Supplementary material for: Involvement of mitogen- and stress-activated protein kinase 1 in BMP-6–induced chondrocyte differentiation
Source: J Biol Chem. 2024 Sep 21;300(11):107806. doi: 10.1016/j.jbc.2024.107806 (PMC11541777; doi:10.1016/j.jbc.2024.107806)
Supplement: Supplemental Fig S1 [file mmc1.docx]

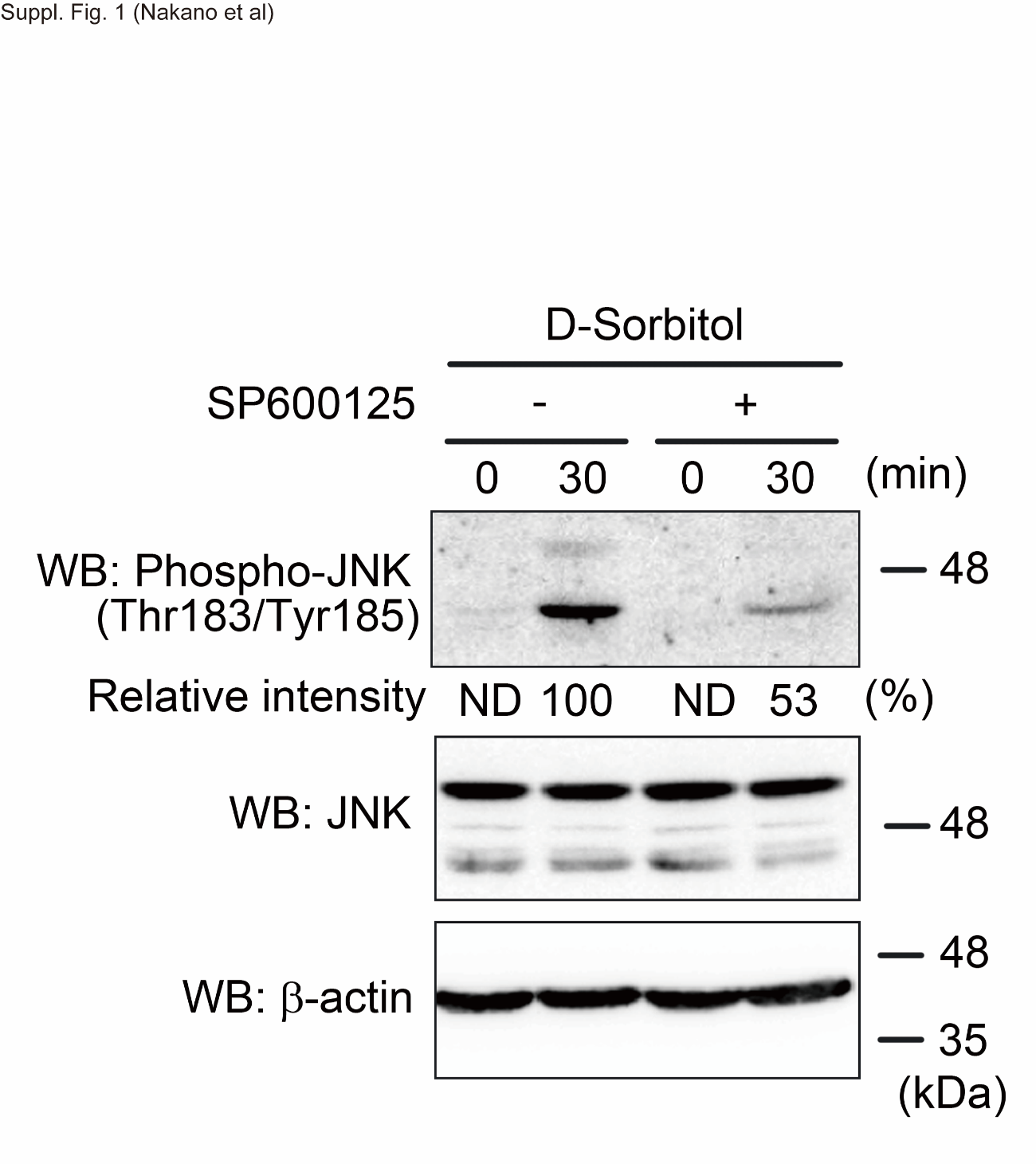


**Suppl. Fig. 1 Phosphorylation of JNK by osmotic stress.** ATDC5 cells were cultured with 0.3 M D-sorbitol for 30 min and then cell lysates were prepared. If necessary, the cells were pretreated with 10 μM SP600125, a JNK inhibitor, for 1 h. The total expression levels of phospho-JNK (Thr183/Tyr185), JNK, and β-actin are indicated in the upper, middle, and lower panels, respectively. Rabbit anti-JNK (#9252) polyclonal antibody was purchased from Cell Signaling Technology. The intensity of the band for phospho-JNK (Thr183/Tyr185) was normalized to the intensity of the band corresponding to JNK. Relative intensity was calculated with respect to cells with D-sorbitol for 30 min.
