# Supplementary material for: Involvement of mitogen- and stress-activated protein kinase 1 in BMP-6–induced chondrocyte differentiation
Source: J Biol Chem. 2024 Sep 21;300(11):107806. doi: 10.1016/j.jbc.2024.107806 (PMC11541777; doi:10.1016/j.jbc.2024.107806)
Supplement: Supplemental Fig S2 [file mmc2.docx]

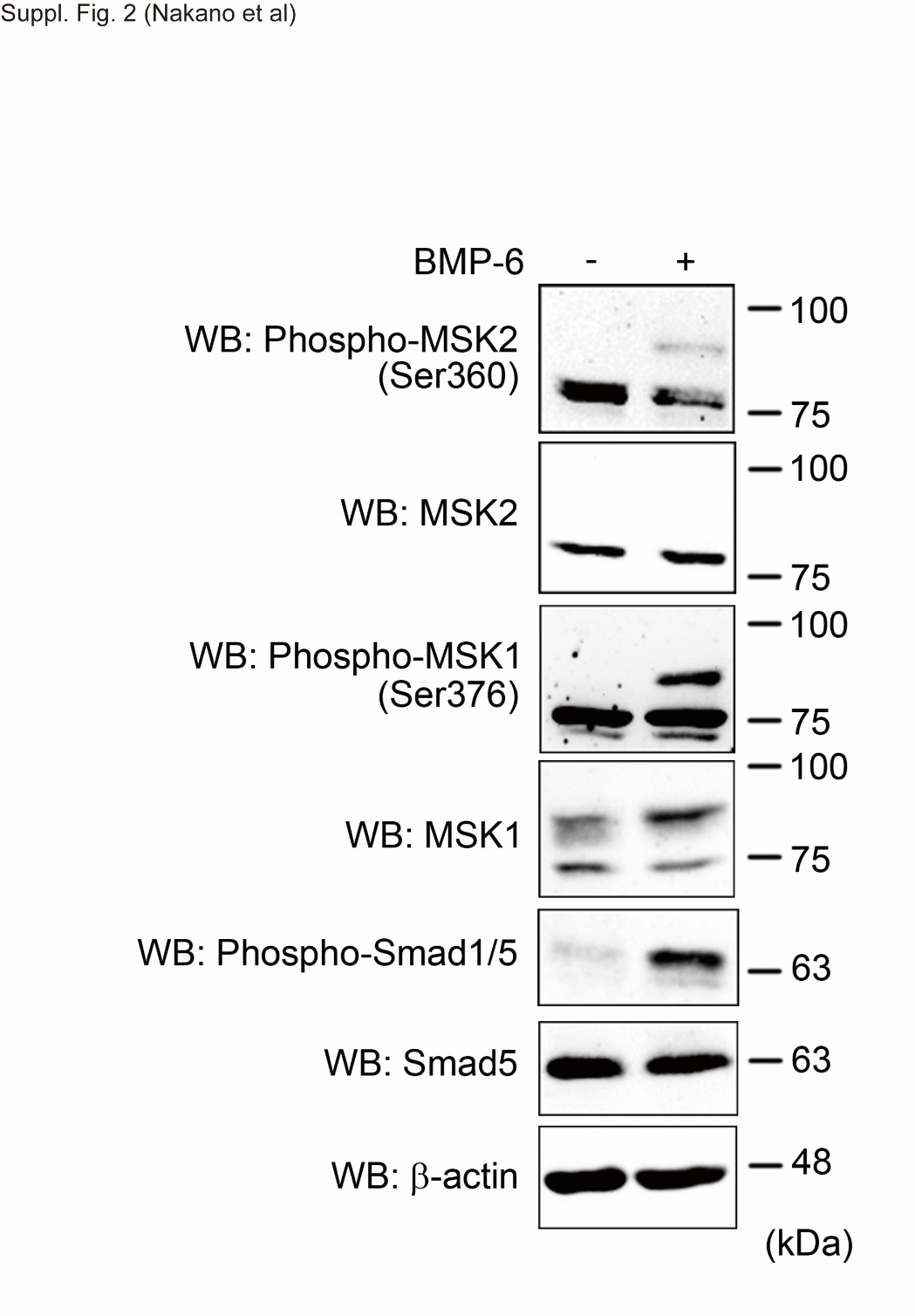


**Suppl. Fig. 2 BMP-6-induced MSK2 phosphorylation in ATDC5 cells.** ATDC5 cells were stimulated with 25 ng/ml BMP-6 for 1 h. The total cell lysates were then used for western blot analyses. The total expression levels of phospho-MSK2 (Ser360), MSK2, phospho-MSK1 (Ser376), MSK1, phospho-Smad1/5, Smad5 and β-actin are indicated in the upper, second, third, fourth, fifth, sixth, and bottom panels, respectively. Rabbit anti-phospho-MSK2 (Ser360) polyclonal (AF7157) and rat anti-MSK2 monoclonal antibodies (MAB2310) were purchased from Affinity Biosciences (Jiangsu, China) and R & D systems (Minneapolis, MN), respectively.
