# Supplementary material for: Involvement of mitogen- and stress-activated protein kinase 1 in BMP-6–induced chondrocyte differentiation
Source: J Biol Chem. 2024 Sep 21;300(11):107806. doi: 10.1016/j.jbc.2024.107806 (PMC11541777; doi:10.1016/j.jbc.2024.107806)
Supplement: Supplemental Fig S3 [file mmc3.docx]

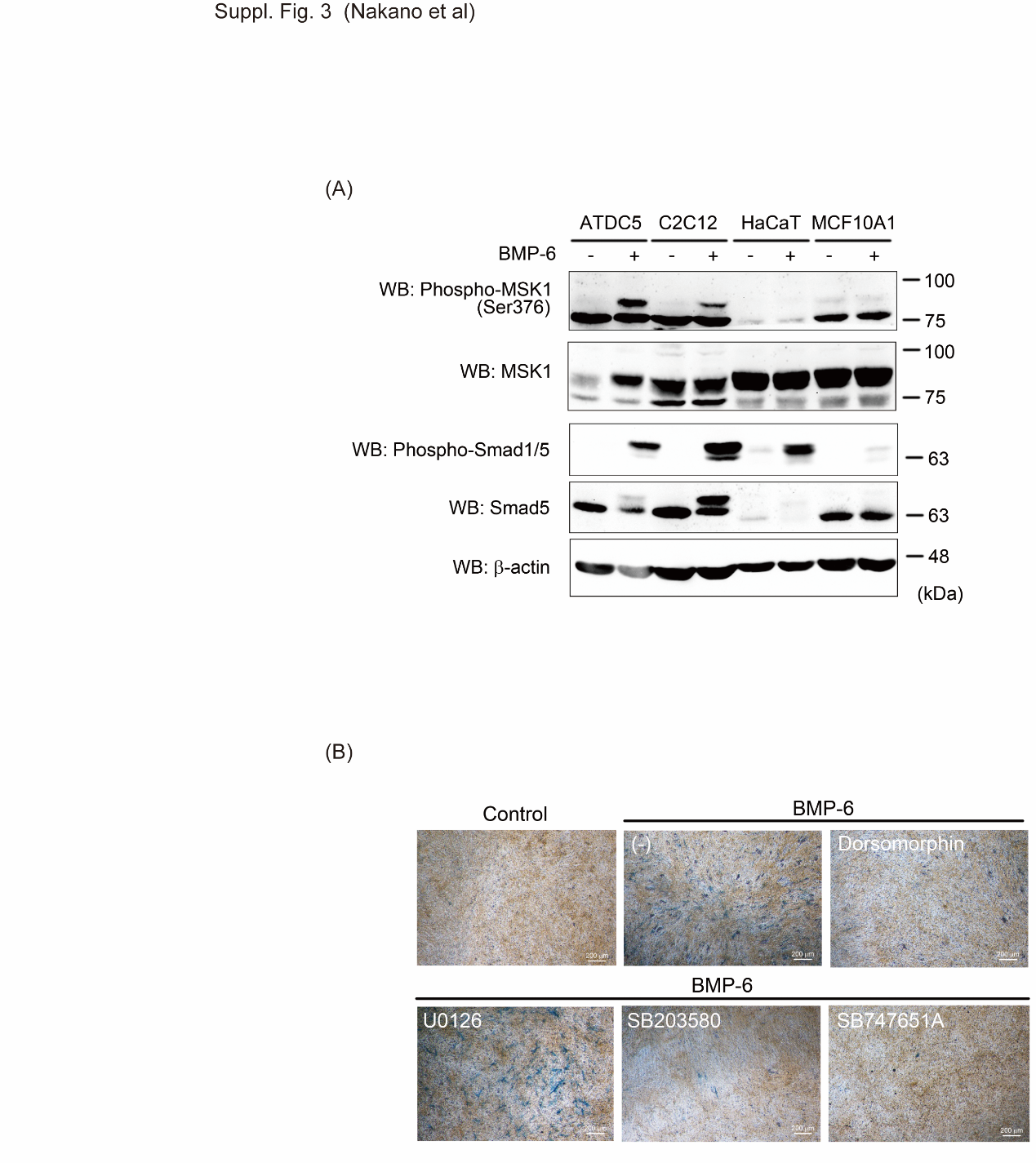
**Suppl. Fig. 3 Requirement of BMP-6-mediated MSK1 activation for osteoblast differentiation in C2C12 cells.** (A) C2C12, HaCaT and MCF10A1 cells, along with ATDC5 cells as a positive control, were stimulated with 25 ng/ml BMP-6 for 1 h. The total cell lysates were then used for western blot analyses. The total expression levels of phospho-MSK1 (Ser376), MSK1, phospho-Smad1/5, Smad5 and β-actin are indicated in the upper, second, third, fourth, and bottom panels, respectively. (B) C2C12 cells were seeded to confluency in a 48-well plate. One day later, the cells were stimulated with 25 ng/ml BMP-6, along with the indicated inhibitors, for 6 days. Afterwards, the cells were stained using an alkaline phosphatase (ALP) staining kit (AK20, Cosmo Bio, Tokyo, Japan). Blue cells indicate ALP-positive osteoblasts (1,2). Scale bar, 200 μm.
