# Supplementary material for: Involvement of mitogen- and stress-activated protein kinase 1 in BMP-6–induced chondrocyte differentiation
Source: J Biol Chem. 2024 Sep 21;300(11):107806. doi: 10.1016/j.jbc.2024.107806 (PMC11541777; doi:10.1016/j.jbc.2024.107806)
Supplement: Supplemental Fig S4 [file mmc4.docx]

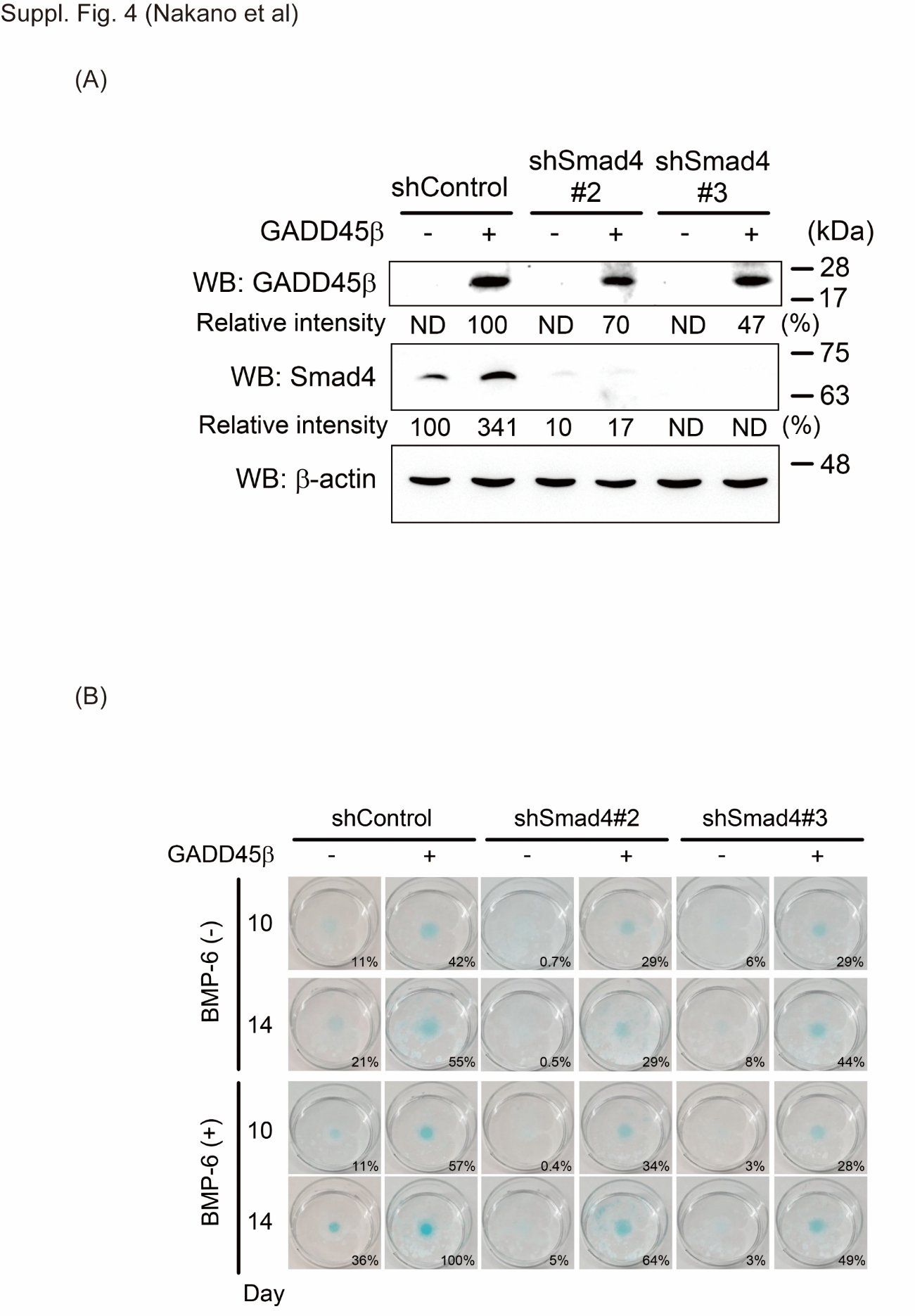
**Suppl. Fig. 4 Effect of GADD45β on BMP-6-mediated chondrocyte differentiation of ATDC5 cells.** (A) GADD45β expression in ATDC5 cells with decreased expression of Smad4 (shSmad4#2 and shSmad4#3 cells). GADD45β was introduced into either shControl, shSmad4#2, or shSmad4#3 cells with a lentiviral vector (pLV-CMV-IRES-puro) (3,4). The total expression levels of GADD45β, Smad4, and β-actin are indicated in the upper, middle, and lower panels, respectively. Rabbit anti-GADD45β polyclonal antibody (GTX46244) was obtained from Gene Tex (Irvine, CA). The intensity of the bands for GADD45β and Smad4 was normalized to the intensity of the bands corresponding to β-actin. Relative intensity was calculated with respect to cells carrying both shControl and GADD45β. (B) Chondrogenic differentiation of GADD45β-expressing ATDC5 cells with decreased Smad4 expression in the presence of BMP-6. After the cells were seeded at a high density, they were stimulated with or without 25 ng/mL BMP-6. Ten (upper and third panels) or 14 days later (second and bottom panels), cells with (third and bottom panels) or without BMP-6 (upper and second panels) were stained with Alcian blue. Representative images are shown. shControl cells were infected with a control lentiviral vector (pLKO.1-TRC-hygro). The intensity of the area stained with Alcian blue was measured using ImageQuant™ TL. The relative intensity of the stained area was calculated with respect to the cells carrying both shControl and GADD45β with BMP-6 for 14 days. ND; not determined.
