# Supplementary material for: Involvement of mitogen- and stress-activated protein kinase 1 in BMP-6–induced chondrocyte differentiation
Source: J Biol Chem. 2024 Sep 21;300(11):107806. doi: 10.1016/j.jbc.2024.107806 (PMC11541777; doi:10.1016/j.jbc.2024.107806)
Supplement: Supplemental Fig S5 [file mmc5.docx]

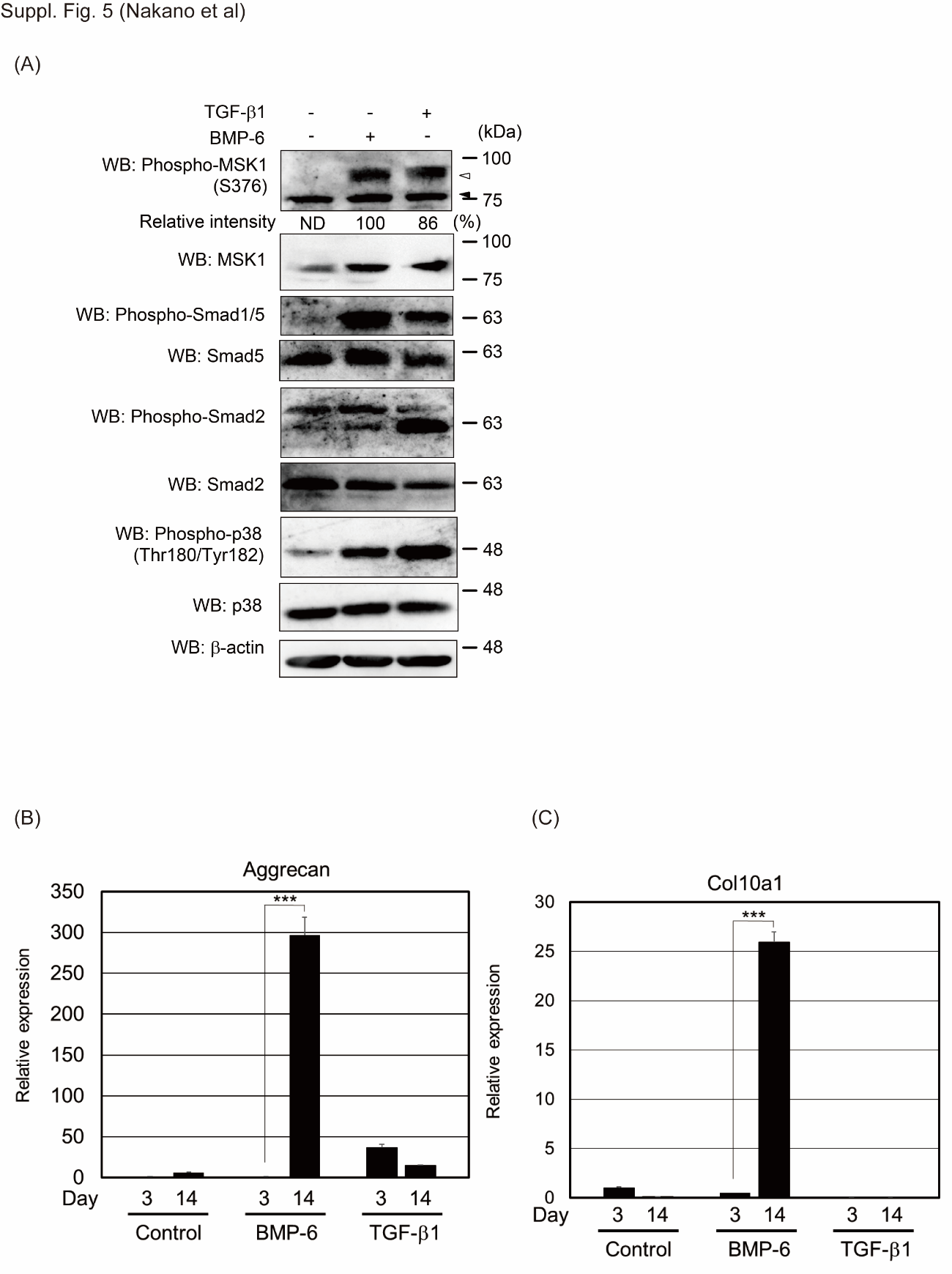


**Suppl. Fig. 5 Effect of TGF-β on the expression of chondrocyte differentiation markers.** (A) MSK1 phosphorylation in ATDC5 cells upon TGF-β stimulation. ATDC5 cells were stimulated with either 5 ng/mL TGF-β1 (cat. no. 100-21, PeproTech) or 25 ng/mL BMP-6 for 1 h, and then total lysates were prepared. Total expression levels of phospho-MSK1 (S376), MSK1, phospho-Smad1/5, Smad5, phospho-Smad2, Smad2, phospho-p38 (Thr180/Tyr182), p38, and β-actin are indicated in the upper, second, third, fourth, fifth, sixth, seventh, eighth, and bottom panels, respectively. Rabbit anti-phospho-Smad2 and anti-Smad2 antibodies are described elsewhere (5-7). White and black arrowheads indicate phospho-MSK1 (Ser376)-specific and-non-specific bands, respectively. The intensity of the band for phospho-MSK1 (Ser376) was normalized to the intensity of the band corresponding to MSK1. Relative intensity was calculated with respect to the 1-h treatment of cells with BMP-6. ND; not determined. (B & C) Expression of aggrecan and collagen10a1 mRNA in ATDC5 cells upon either TGF-β1 or BMP-6 stimulation. The cells were cultured at a high density and then stimulated with 5 ng/mL TGF-β1 or 25 ng/mL BMP-6. Three or 14 days later, total mRNAs were prepared for qPCR to detect aggrecan (B) and collagen10a1 mRNAs (C). All values represent means ± SDs (n = 3). Asterisks indicate significant differences.
